# Physiologically Based Pharmacokinetic Modelling of Cabozantinib to Simulate Enterohepatic Recirculation, Drug–Drug Interaction with Rifampin and Liver Impairment

**DOI:** 10.3390/pharmaceutics13060778

**Published:** 2021-05-22

**Authors:** Bettina Gerner, Oliver Scherf-Clavel

**Affiliations:** Institute for Pharmacy and Food Chemistry, University of Würzburg, 97074 Würzburg, Germany; bettina.gerner@uni-wuerzburg.de

**Keywords:** physiologically based pharmacokinetic (PBPK) modeling, Cabozantinib, enterohepatic recirculation, drug–drug interactions (DDIs), liver impairment, cytochrome P450 3A4 (CYP3A4), pharmacokinetics

## Abstract

Cabozantinib (CAB) is a receptor tyrosine kinase inhibitor approved for the treatment of several cancer types. Enterohepatic recirculation (EHC) of the substance is assumed but has not been further investigated yet. CAB is mainly metabolized via CYP3A4 and is susceptible for drug–drug interactions (DDI). The goal of this work was to develop a physiologically based pharmacokinetic (PBPK) model to investigate EHC, to simulate DDI with Rifampin and to simulate subjects with hepatic impairment. The model was established using PK-Sim^®^ and six human clinical studies. The inclusion of an EHC process into the model led to the most accurate description of the pharmacokinetic behavior of CAB. The model was able to predict plasma concentrations with low bias and good precision. Ninety-seven percent of all simulated plasma concentrations fell within 2-fold of the corresponding concentration observed. Maximum plasma concentration (C_max_) and area under the curve (AUC) were predicted correctly (predicted/observed ratio of 0.9–1.2 for AUC and 0.8–1.1 for C_max_). DDI with Rifampin led to a reduction in predicted AUC by 77%. Several physiological parameters were adapted to simulate hepatic impairment correctly. This is the first CAB model used to simulate DDI with Rifampin and hepatic impairment including EHC, which can serve as a starting point for further simulations with regard to special populations.

## 1. Introduction

Tyrosine kinase inhibitors (TKI) play an increasingly important role in the therapy of multiple malignancies and the development of compounds targeting, for example, the vascular endothelial growth factor (VEGF)/VEGF receptor (VEGFR) signaling pathway has led to key advances in the treatment of different cancer types [1,2,3]. One representative of this drug class is the multi-target TKI Cabozantinib (CAB), which is currently approved for the treatment of metastatic medullary thyroid cancer (MTC) [4,5], advanced renal cell carcinoma (RCC) [6,7] and hepatocellular carcinoma (HCC) [8,9]. The primary targets of CAB are MET (hepatocyte growth factor receptor, HGFR) and VEGFR2 (vascular endothelial growth factor receptor 2), which are both important mediators of tumor growth and tumor angiogenesis [10]. In addition to approved indications, CAB is currently investigated in numerous clinical trials, covering a wide range of different cancer types [11]. This could lead to approval in other types of cancer and to additional patient populations being treated with CAB. Thus, it is important to gain a deeper knowledge of the pharmacokinetic (PK) behavior of CAB to ensure the most effective and safe therapy for a broad range of patients. CAB is orally administered once daily either as capsule (Cometriq^®^) or tablet formulation (Cabometyx^®^). Maximum plasma concentrations (C_max_) after a single dose occur approximately at 3 to 5 h post-dose. CAB shows a long terminal half-life (~120 h), is highly bound to human plasma proteins (99.7%) and undergoes extensive metabolism as it is a substrate of Cytochrome P450 (CYP3A4) [12]. Four major CAB metabolites can be found in plasma: Exel-5366 (Amide Cleavage Product), Exel-1644 (6-Desmethyl Amide Cleavage Product Sulfate), Exel-1646 (Monohydroxy Sulfate) and Exel-5162 (N-Oxide). An illustration of the chemical structure of CAB and its main metabolites is shown in the Electronic Supplementary Material (ESM) Figure S1. The hepatobiliary elimination seems to be the predominant route of excretion in humans, whereas urinary excretion is only relevant for the CAB metabolites. Metabolites predominantly found in urine were dequinolinyl CAB glucuronide, dequinolinyl CAB sulfate and EXEL-5366 (CAB amide cleavage product) [13].

CAB plasma concentration time profiles are characterized by a rapid decrease in CAB plasma concentration in the first hours followed by a long terminal half-life. In addition, PK studies in human and rats revealed multiple peaks in the plasma concentration time profiles starting approximately 24 h after CAB administration. This phenomenon is most likely due to an enterohepatic circulation (EHC), but not finally confirmed yet [12,14,15], as also other causes, like absorption in deeper bowel sections or the irregular pattern of gastric emptying, might be conceivable [16,17].

CAB is susceptible to drug–drug interactions (DDI), as it is mainly metabolized via CYP3A4. The combination of CAB with Rifampin (RIF), a strong CYP3A4 inducer, resulted in increased CAB clearance (4.3-fold) and reduced CAB exposure (AUC) by 77% [18]. Therefore, inducers like Rifampin, St. John’s Worth, Phenytoin, Carbamazepine or Phenobarbital should be avoided according to the EMA summary of product characteristics (SPC) [19]. In contrast to that, the FDA label does not make a general recommendation to avoid the combination but recommends increasing the daily CAB dose by 20 mg (Cabometyx^®^) and 40 mg (Cometriq^®^), respectively. As the hepatobiliary pathway appears to be the main route of elimination for CAB, hepatic dysfunction may have a relevant influence on CAB plasma concentrations. Hepatic diseases can occur in relevant patient populations due to co-medication and disease (e.g., HCC and liver metastases) and is characterized by a loss of functional hepatocytes, resulting in possibly toxic or ineffective plasma concentrations. To investigate different liver disease states, one aim was to expand the PBPK model to patients with mild and moderate liver impairment.

PBPK approaches provide a framework to combine information on physiology, population and drug characteristics, and to extract maximal information from available data to gain a mechanistic understanding of key processes in the PK of a drug [20,21,22,23]. One strength of PBPK modelling is the possibility to gain mechanistic insights into pharmacokinetics and in vivo behaviour of a compound by using it as a simulation tool where the focus is less on the quantitative predictions but much more on hypothesis generation and testing [21,24]. Therefore, the aim of this work was to investigate the hypothesis of EHC and to describe the PK of CAB. The PBPK model was also used as a tool to simulate the concomitant administration of RIF and the influence of liver impairment regarding changes in plasma concentration time profiles and drug exposition.

## 2. Materials and Methods

### 2.1. Software

The PBPK model was built using PK-Sim^®^ version 8 as part of the open source modeling software Open Systems Pharmacology Suite (Bayer Technology Services, Leverkusen, Germany) [25]. For a detailed insight into the software, the input and output parameter as well as the description of the generic model structure of PK-Sim^®^ refer to Eissing et al. [26], Willmann et al. [27], Kuepfer et al. [28] or the user manual [29]. All published study data were digitized using WebPlotDigitizer (Version 4.3, Ankit Rohatgi, Pacifica, CA, USA). Microsoft Excel 2016 Version 16.0 (Microsoft Corporation, Redmond, WA, USA) and R Studio Version 1.1.383 (RStudio Incorporation, Boston, MA, USA) running R version 3.6.3 (R Foundation for Statistical Computing, Vienna, Austria, 2020) [30] were used for model performance, statistical calculations and plot generation.

### 2.2. Clinical Data

Human peroral data from the mass balance study, one bioequivalence study (CAB capsule vs. tablet), one dose proportionality study (20, 40, 60 mg tablet), a DDI study (divided in a Rifampin and Ketoconazole arm), a food effect and a PPI (proton pump inhibitor) effect were digitized and used for model development and evaluation. The model development process was supplemented with intravenous (iv) (5 mg/kg, 10 mg/kg) data from rats, published by Wang et al. [2]. The gathered CAB peroral plasma profiles were split into a training dataset, used for model development and parameter optimization, and a test dataset for model evaluation. Data from the liver impairment study were used to investigate the ability of the model to simulate different hepatic disease states. All human studies used a single dose of either CAB solution, tablet, or capsule covering a dose range from 20 to 140 mg, depending on the study design. A summary of each study regarding the demographics, administration protocols and the allocation to either the training or the test dataset, respectively, or to the DDI and hepatic impairment simulations is given in Table S1 of the ESM. Sampling times of each study are presented in Table S2.

### 2.3. Workflow and Model Development

The PBPK modelling was performed in a stepwise procedure. Rat iv data were used for the development of an initial PBPK model. All physicochemical data of CAB found through an intensive literature search were used as input parameters. Different methods for the calculation of tissue distribution and cellular permeability were evaluated [31]. The generic rat individual, which is already an integral part of PKSim^®^ was adapted according to the animals used by Wang et al. and implemented in the model. In a second step, simulations for rat intragastric (ig) administration were performed. All input values of the iv model were transferred to the ig model. Relevant input parameters and knowledge from the rat PBPK models were used to build a first human PBPK model. For model building in humans a virtual mean individual according to the demographics of the respective studies mentioned in Table S1 was created and used in these simulations. To account for the second peak in the plasma concentration time profiles, the fast decrease in CAB plasma concentration in the approximately first 20 h, and the long terminal half-life different hypothesis were tested. Different CAB formulations (solution, tablet, and capsule) were investigated separately. For further model development and evaluation, virtual populations containing 100 individuals were created. An implemented PKSim^®^ algorithm was used to generate a variation of anthropometric parameters within the limits of the ICRP or NHANES database [28,29], respectively. These parameters were kept within the limits of the respective study population. Parameters which were not found in the literature were estimated and single parameters were adjusted according to the observed data. Local sensitivity analysis to evaluate the influence of changes in input parameters on the final PBPK model were conducted, especially if the parameter had been optimized or might have had a strong influence on the model due to the calculation methods in PK-Sim^®^. Data from the DDI study of CAB with RIF were simulated to verify the implemented CYP3A4 process. To simulate CAB plasma concentration time profiles in mild and moderate hepatic impairment, relevant physiological parameters of the final PBPK model were adapted. A workflow of the model development and evaluation process is given in the ESM (Figure S2). Data of all included rat and human studies are shown in different plots in the ESM (Figures S3–S6). A more detailed description of the observed human and rat plasma concentration time profiles and further information on the model development process can also be found in the ESM.

### 2.4. Enterohepatic Circulation Modelling

A gallbladder emptying process was implemented in PKSim^®^ and relevant physiological parameters, like the emptying half-time were optimized. To enable biliary excretion and EHC, substrate affinity towards a canalicular efflux transporter like MRP2 (multidrug resistance-associated protein 2) is a prerequisite. MRP2 is an efflux transporter which is predominantly located on the canalicular membrane of the hepatocytes [32] and was included into the model according to the PK-Sim^®^ human gene expression databank because CAB has been found to be a substrate of this transporter [13]. As an assumption, the gallbladder emptying process was activated three times over 24 h, as this process is related to food intake, which was considered to take place in the morning, in the noon and in the evening [33]. A fasted state, meaning no active gallbladder emptying, was assumed for the first hours, as all studies were conducted under fasted conditions.

### 2.5. Model Evaluation

To evaluate the predictive performance of the model, multiple methods were used [20,34,35]: Visual inspections between observed and simulated plasma concentration time profiles were carried out initially. In addition, predictive performance of the PBPK model was analysed by generating goodness-of-fit plots in which the predicted plasma concentrations was plotted against their corresponding observed values according to the EMA guideline on the reporting of PBPK modelling and simulation [34]. Predicted maximum concentration (C_max_) and the predicted area under the systemic drug concentration–time curve from time zero to the time of the last concentration (AUC_last_) were compared to the respective literature values. Model accuracy and precision was described by using prediction error (PE), mean prediction error (MPE) and mean absolute prediction error (MAPE). The calculations were made according to Equations (1)–(3).

(1) $$\mathrm{PE}\;\left[\%\right]=\frac{C_{predicted,i}-C_{observed,i}}{C_{observed,i}}\;\cdot \;100\%$$

(2) $$\mathrm{MPE}\;\left[\%\right]=\frac{1}{n}\;\;\overset{n}{\underset{i=1}{\sum }}\mathrm{PE}i$$

(3) $$\mathrm{MAPE}\;\left[\%\right]=\frac{1}{n}\;\;\overset{n}{\underset{i=1}{\sum }}\left|\mathrm{PE}i\right|$$

Mean relative deviation (MRD, Equation (4)) of the predicted and observed plasma concentrations was calculated for a quantitative measure of model performance. MRD values ≤2 characterize an adequate model performance and therefore were considered acceptable.

(4) $$\mathrm{MRD}=10^{x};x=\sqrt{\frac{1}{n}\overset{n}{\underset{i=1}{\sum }}{\left(log_{10}c_{predicted,i}-log_{10}c_{observed,i}\right)}^{2}}$$

Abbreviations in Equations (1)–(4) are as follows: *c_predicted,i_* = predicted plasma concentration, *c_observed,i_* = corresponding observed plasma concentration, *n* = number of observed values.

### 2.6. DDI Interaction between CAB and RIF

The developed CAB PBPK model was combined with the Rifampicin PBPK model, developed by Hanke et al. to simulate DDI between CAB and RIF [36]. Clinical study data from a DDI interaction study between CAB and RIF were used and the interaction between CAB and RIF was recreated in silico. Multiple dosing simulations for capsule and tablet administration were conducted to evaluate the impact of concomitant RIF medication. For the administration of CAB together with RIF higher CAB daily doses were used (180 mg capsule; 80 mg tablet), according to the FDA recommendation. Regular doses (140 mg capsule; 60 mg tablet) once daily were maintained if CAB was given alone. The quality of the DDI interaction modelling was evaluated by comparison of the respective plasma concentration time profile and through calculation and comparison of the ratios of AUC_last_ (Equation (5)) and C_max_ (Equation (6)) for the administration of CAB alone or together with the perpetrator.

(5) $${\mathrm{DDI}\;\mathrm{AUC}}_{\mathrm{last}}\;ratio=\frac{{\mathrm{AUC}}_{\mathrm{last}}\;\mathrm{CAB}\;in\;combination\;with\;\mathrm{RIF}}{{\mathrm{AUC}}_{\mathrm{last}}\;\mathrm{CAB}\;alone}$$

(6) $${\mathrm{DDI}\;C}_{\max}\;ratio=\frac{C_{\max}\;\mathrm{CAB}\;in\;combination\;with\;\mathrm{RIF}}{C_{\max}\;\mathrm{CAB}\;alone}$$

### 2.7. Hepatic Impairment Simulations

For the hepatic impairment simulations, individuals and populations (*n* = 100) with demographic properties according to the respective study data [37] were created. Several physiological changes are relevant in patients with hepatic impairment and were adapted accordingly [38,39,40]: The portal liver blood flow and the renal blood flow were reduced, the liver blood flow and the blood flows for the remaining organs, except for the brain, were increased according to Edginton et al. [38]. For the enzyme-specific clearance, the activity of CYP3A4 was adapted also based on Edginton et al. and reduced liver volume fraction and different hematocrit values for mild and moderate liver impairment were used. The plasma protein scale factor, which is part of the structural model in PKSim^®^, was adapted to describe changes in the plasma protein concentration and protein binding, respectively. All simulations were performed based on a single administration of 60 mg CAB as capsule formulation. Evaluation of model performance was conducted via visual inspection of the plasma concentration time profile and by comparison of the predicted and observed plasma exposure and C_max_ of the healthy control group versus patients with mild and moderate liver impairment, respectively.

## 3. Results

### 3.1. Rat Intravenous and Intragastric Simulations

**Figure 1:**
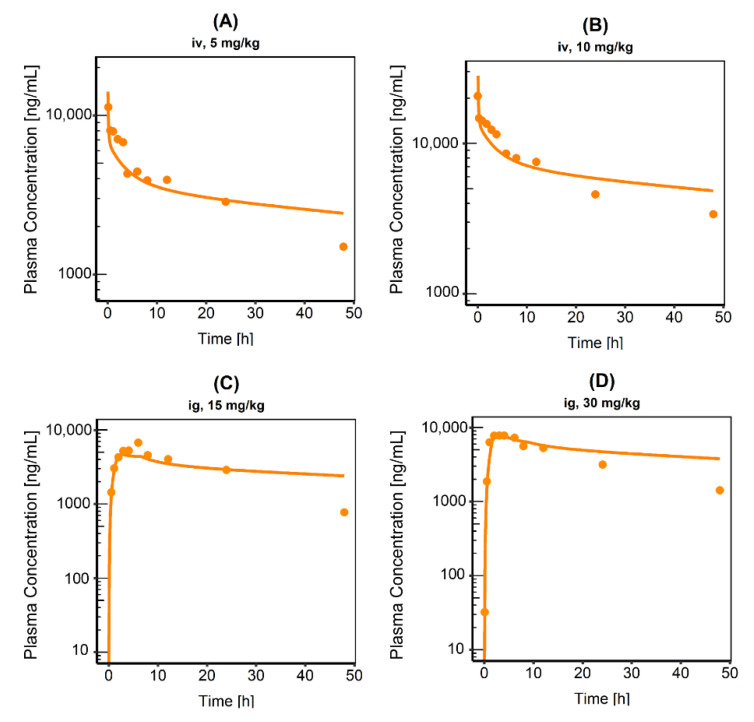
Semi-logarithmic plots of plasma concentration time profiles following (**A**) 5 mg/kg intravenous (iv), (**B**) 10 mg/kg iv, (**C**) 15 mg/kg intragastric (ig) or (**D**) 30 mg/kg ig administration of CAB. Observed data are shown as dots, simulations are shown as lines. All data extracted from Wang et al., *n* = 8 in every case.

The first simulations were performed based on iv data and physicochemical values, which were extracted from the literature. The best results for the calculation of tissue distribution and cellular permeability were obtained for a combination of Rodgers & Rowland and the PKSim^®^ standard method. As no further information on specific processes, like CYP metabolism or MRP2 affinity, was available, a total hepatic clearance was assumed as a surrogate and optimized based on the in vivo data [41]. Afterwards, all input values of the iv model were transferred to the ig model. As for the first ig simulations there was an overestimation in plasma concentration, intestinal permeability and gastrointestinal solubility were optimized to describe the observed data more precisely. The value for plasma protein binding was adopted from humans, as in general there is a good agreement between the protein binding in human and rat plasma [42]. This was also reported for EXEL-1644, which has a similar protein binding in rats (99.729% to 99.966%) compared to humans (99.950% to 99.996%) [13]. The final parameters used for the rat PBPK model are shown in Table S3. The iv and the ig model were both able to describe the plasma concentration time profiles of CAB in rats (Figure 1) and showed a high accuracy illustrated by a low bias (MPE range −6.4% to +12.2%) and a good precision (MAPE range 18.4–33.8%). A MRD of all predicted plasma concentrations ≤2 was achieved in all simulations (MRD range 1.27–1.63). Table S4 summarizes the respective PK parameters C_max_ and AUC_last_ as well as the values for MPE, MAPE and MRD.

### 3.2. Human Peroral Simulations for Different CAB Formulations

All drug dependent parameters, e.g., lipophilicity or molecular weight, should be the same across species and were transferred from the rat to the human model. Calculation methods for distribution and cellular permeability should also be the same across different species. Several calculation methods were tested during model development. Best results were also obtained for the Rodgers & Rowland tissue distribution in combination with the PKSim^®^ standard method for the calculation of cellular permeabilities. Parameters, which could not be transferred from the rat simulations, were estimated. These included EHC parameter (gallbladder ejection half-time, time to complete gallbladder refilling, EHC continuous fraction, gallbladder ejection fraction), as well as k_cat_ and K_M_ for MRP2. In vitro values for k_cat_ and K_M_ for CYP3A4 were available from a study by Lin et al. [43]. However, the purpose of that study was to elucidate the enzymatic characteristics of different CYP3A4 alleles in vitro. Their K_m_ value was used more like an internal comparison for different isoenzymes and was determined under non-physiological conditions without taking protein-binding into account, representing rather the free K_m_. Both in vitro values were therefore checked and adapted to fit the data and especially for K_m_ there was a high deviation from the reference value for the reasons stated. Renal, hepatic, and biliary excretion were investigated, but no renal excretion was found for CAB, which is consistent with literature [13]. As a CYP3A4 metabolism and an active MRP2 transport were used in the human model, no additional liver and biliary plasma clearance was implemented. Different CAB formulations (solution, tablet, and capsule) were investigated separately, as they are not bioequivalent. The formulation type “dissolved” was used for the oral solution. This formulation type characterizes the drug as being in solution at the point of oral administration. However, there was no further information on the administered solution in the original study (e.g., additional solubiliser) and as CAB is a poorly soluble drug, it might be possible that the given solution was rather a suspension than a real solution. Therefore, dissolution at the point of administration might be incomplete and the intestinal absorption may be limited by the solubility, imposing an upper bound to the absorption rate. To mimic the possibly biased solubility and the overestimation of absorbed substance, the gastrointestinal solubility was reduced, just as it was done in the oral simulations for rats. Nevertheless, there was still some overestimation of the CAB plasma concentration, especially for later time points. For the simulation of CAB tablets and capsules, a Weibull function was used to create a tablet and capsule formulation, respectively. All relevant parameter of the Weibull function were adapted to fit the observed data. All parameters used in the final PBPK model are shown in Table 1.

**Figure 2:**
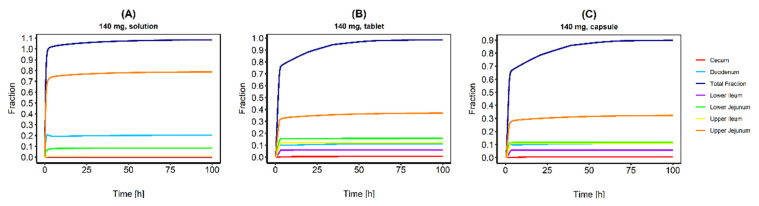
Simulation of the absorbed CAB fractions in different intestine sections after administration of a CAB 140 mg solution (**A**), tablet (**B**) and capsule formulation (**C**).

**Figure 3:**
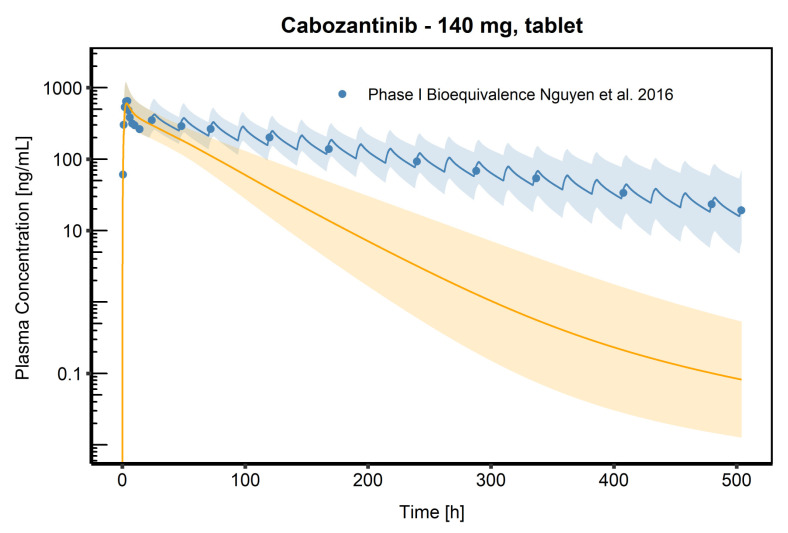
Comparison of simulated CAB venous blood plasma concentration time profiles (semi-logarithmic) following oral administration of 140 mg CAB (tablet formulation). The blue shaded area represents the geometric mean SD for population simulations with an implemented EHC process. The orange shaded area represents the geometric mean SD for population simulations without an implemented EHC process. Geometric means are shown as blue line (with EHC), respectively, as orange line (without EHC). Observed data are shown as blue dots.

The fractions absorbed in different intestine sections were simulated after administration of a CAB 140 mg solution, tablet, and capsule (Figure 2). In case of the solution, the total fraction absorbed was reached almost instantaneously and the complete dose was absorbed. For the tablet formulation, only about 75% of the dose was absorbed in the first three hours and it took about 30 h until 95% of the dose was absorbed. For the capsule formulation, a slightly lower fraction was absorbed in the first three hours (65%), which agrees with the lower C_max_ compared to the tablet formulation. After 30 h, about 82% of the given dose was absorbed, conforming to the lower relative bioavailability compared to the solution (74–93% for capsule; 97% for tablet) [12]. Plasma concentration time profiles with and without an integrated EHC after the administration of a 140 mg tablet process are compared in Figure 3. If no EHC process was included, the observed plasma concentrations were significantly underestimated, and CAB plasma exposure was 2.7 times lower compared to the plasma exposure with an included EHC (23,809.01 ng/h/mL vs. 64,750.20 ng/h/mL).

### 3.3. PBPK Model Evaluation

**Figure 4:**
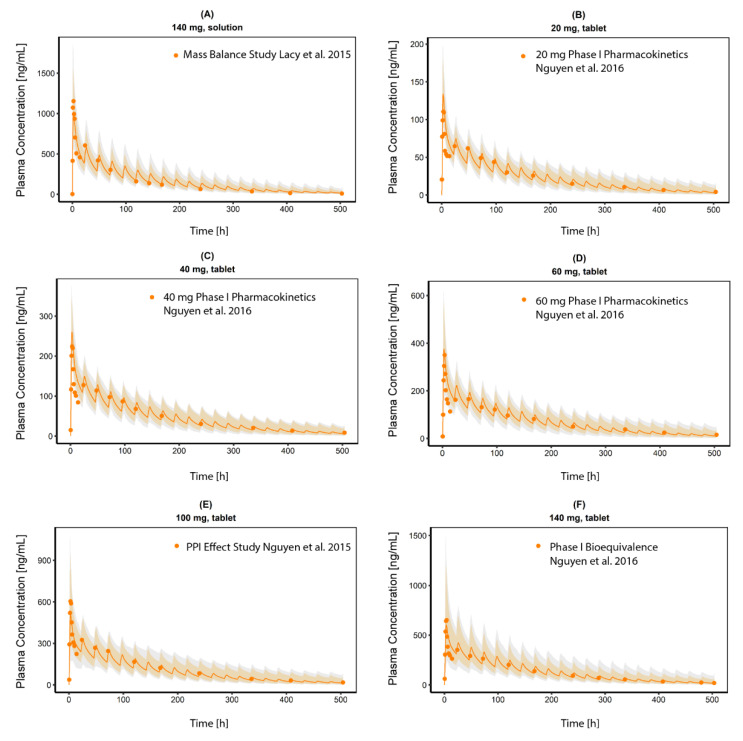

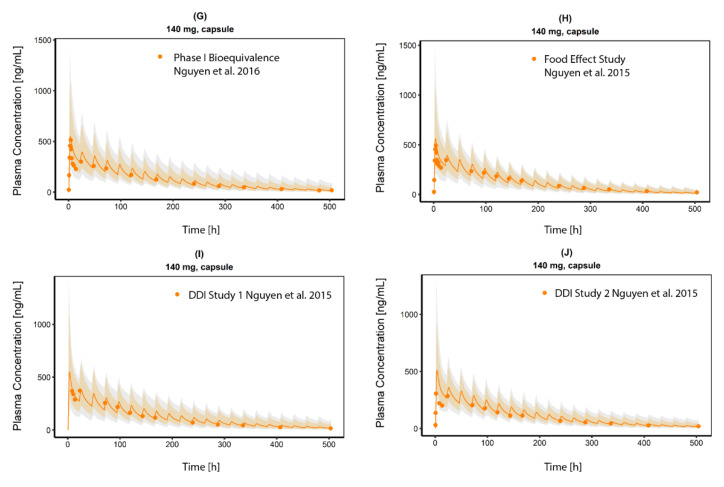
CAB plasma concentration time profiles (linear). (**A**) 140 mg CAB oral solution from a mass balance study, (**B**–**D**) data from a Phase I study CAB as tablet in 20, 40 and 60 mg oral single dose, (**E**) CAB 100 mg oral single dose as tablet in a PPI effect study, (**F**,**G**) 140 mg CAB as tablet an capsule formulation from a bioequivalence study, (**H**) 140 mg CAB oral single dose as capsule formulation from a food effect study, (**I**,**J**) 140 mg CAB oral single dose as capsule formulation from DDI studies with Ketoconazole and Rifampin. Observed data are shown as orange dots. Population simulation (*n* = 100) geometric means are shown as orange lines; the shaded orange areas represent the predicted population geometric SD. The shaded grey areas represent the 5% to 95% prediction interval.

**Figure 5:**
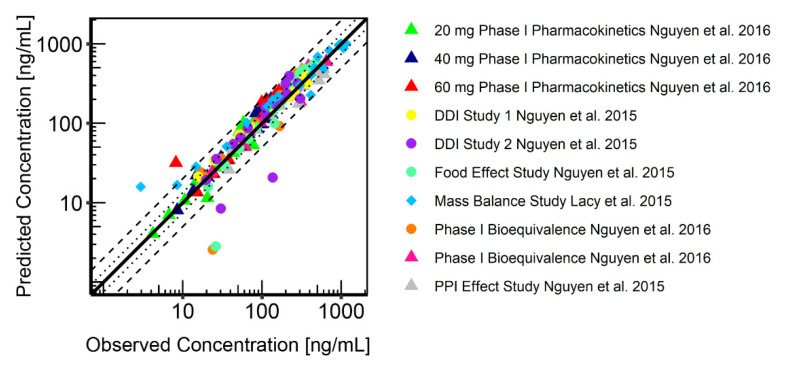
Goodness-of-fit plot showing predicted (geometric mean) versus observed CAB plasma concentrations. Tablet formulations are represented by triangles, capsule formulations are represented by dots, the solution is represented by diamonds. The black solid line represents the line of identity; dashed black lines represent a twofold deviation; dotted black lines represent a 1.25-fold deviation.

**Figure 6:**
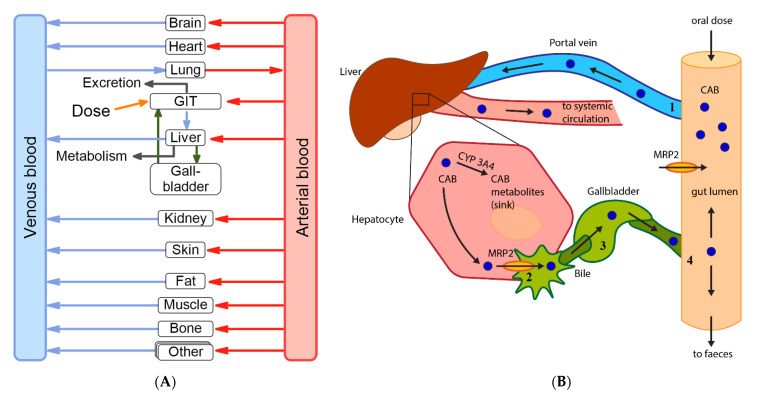
(**A**) Overview of the final PBPK model. Cabozantinib enters the PBPK model via oral administration to the GIT and is eliminated by metabolism and faeces (see (**B**) for further details). Cabozantinib metabolites were not modelled due to insufficient data. *GIT:* gastro-intestinal-tract (**B**) Schematic illustration of the EHC model of Cabozantinib after oral drug administration: (**1**) after intestinal absorption, CAB is distributed via the hepatic portal vein into the liver; (**2**) once in the liver, a fraction of CAB is excreted into the bile via MRP2 transporter; (**3**) bile is transferred into the gallbladder where it is stored and concentrated; (**4**) after a given accumulation time, bile is delivered back into the intestine and a fraction of recirculated CAB can be reabsorbed again in the intestine or eliminated with faeces. To model EHC of the drug, active transport processes via MRP2, storage in gallbladder, secretion into the duodenum and reabsorption along the intestine are included in the model. Elimination occurs mainly via hepatic and intestinal (not shown for simplicity) metabolism (CYP 3A4) and faeces. *CAB*: Cabozantinib; *MRP2*: multidrug resistance-associated protein 2.

The final PBPK model was successfully used to describe observed CAB plasma concentrations in healthy volunteers (HVs) after a single oral dose. Simulated plasma profile trajectories were in close concordance with observed data. Linear plots of predicted versus observed plasma concentration time profiles are shown in Figure 4. Semi-logarithmic plots can be found in Figure S7 of the ESM. Figure 5 shows the goodness-of-fit plots comparing predicted to observed plasma concentrations. Ninety-seven percent of all simulated plasma concentrations fall within 2-fold of the corresponding concentration observed. Figure S8 shows the predicted vs. observed area under the concentration time curves from the first to the last data point (AUC_last_) and maximum plasma concentration (C_max_) values of all studies. All predicted AUC_last_ and C_max_ values fell within the 2-fold acceptance criterion. Ratios for predicted versus observed AUC_last_ values are between 0.9 and 1.2 and for C_max_ between 0.8 and 1.1. All values can be found in the ESM (Table S5). Results for model bias (mean prediction error), model precision (mean absolute prediction error) and MRD are listed in Table S6. Mean MRD was 1.53 with a range of 1.22 to 1.88, thus all fulfilled the acceptance criterion. Results of the local sensitivity analysis, which was performed based on the simulation of the 140 mg CAB capsule administration, are demonstrated in Figure S9. For a graphical representation of the final model including a detailed view on the EHC, see Figure 6.

### 3.4. Simulations of DDI between CAB and RIF

**Figure 7:**
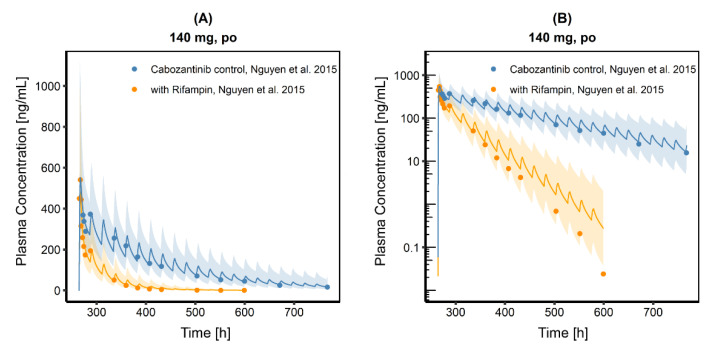
Cabozantinib plasma concentration time profiles in linear (**A**) and semi-logarithmic (**B**) representation after a single oral dose of 140 mg Cabozantinib (capsule formulation) without and with rifampin co-administration. Observed data are shown as dots. Population simulation (*n* = 100) geometric means are shown as lines; the shaded areas represent the predicted population geometric SD.

The plasma concentration time profile after single-dose administration of 140 mg CAB capsule, given either alone or together with its perpetrator RIF, could be described adequately (Figure 7). There is a tendency to overestimate CAB plasma concentration for later time-points (>300 h); however, except for the last value, all observed concentrations were within the geometric mean SD. The ratio for AUC_last_ of CAB administered together with RIF and CAB administered without RIF was 0.23, which is a reduction in AUC_last_ by 77%. This agrees with the reduced CAB exposure (77%) found by Nguyen et al. [18], which is also stated in the EMA and FDA SPC and label for Cabometyx^®^ and Cometriq^®^. The DDI C_max_ ratio was 87.0%, which is within 80–125% and, therefore, indicates that RIF co-administration does not relevantly influence CAB absorption (Table 2).

Multiple dosing simulations for CAB tablet (60 mg) and capsule (140 mg), given without RIF, resulted in predicted average steady state plasma concentrations (C_ss_) of 1197.44 ng/mL, respectively, 1576.68 ng/mL. Simulations after multiple-dose administration of CAB together with RIF and an increased CAB dose by 20 mg (tablet), respectively 40 mg (capsule) led to C_ss_ of 394.74 ng/mL and 532.41 ng/mL. Thus, even with higher CAB doses, DDI C_ss_ were still about two-thirds lower compared to the C_ss_ if CAB is given without its perpetrator. Table S7 gives a clearer presentation of all values. Figure S10 shows predicted Css either for CAB capsule or tablet given alone (CAB standard dose) or together with 600 mg RIF (increased CAB dose).

### 3.5. Investigation of Hepatic Impairment on CAB Plasma Exposure

**Figure 8:**
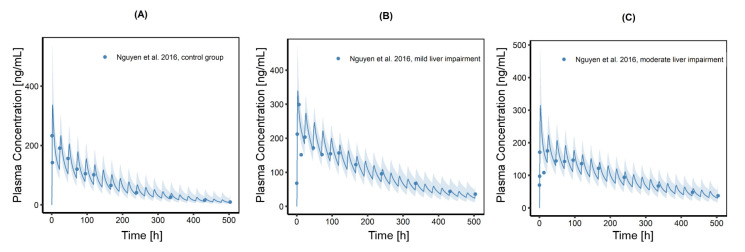
Population simulations (*n* = 100) of CAB plasma concentration time profiles after a single dose of 60 mg CAB capsule to (**A**) the healthy control group, (**B**) patients with mild liver impairment and (**C**) patients with moderate liver impairment. Blue dots indicate mean observed plasma concentrations, the blue line represents the population simulations geometric mean of the predicted plasma concentrations; the shaded areas represent the predicted population geometric SD.

Individuals and populations with mild and moderate liver impairment were created and the model was able to describe CAB plasma exposure in the healthy control group as well as in the diseased populations. An overview of the final physiological parameters changed for mild and moderate liver impairment in comparison to the healthy control group is given in Table 3. Figure 8 shows predicted plasma concentration time profiles for these populations (*n* = 100) including observed CAB plasma concentrations. In Figure S11 of the ESM, the trajectories are compared in one plot, indicating the higher plasma exposure in mild and moderate liver impairment compared to the control group. AUC_last_ for patients with mild and moderate hepatic impairment was increased by 64% and 50%, respectively (51,633 ng/h/mL for mild impairment and 47,096 ng/h/mL for moderate impairment vs. 31,448 ng/h/mL for the control group). Mean predicted C_max_ is slightly increased in the mild impairment group and slightly lowered in the moderate impairment group. Patients in the mild liver impairment group of the underlying study had a minimal lower fraction of unbound CAB in plasma (fu_p_) compared to the control group, whereas patients in the moderate liver impairment group had a higher fu_p_ compared to the control group. To include this observation, a plasma protein scale factor, which has a strong influence on C_max_ and AUC_last_, was adapted to describe the plasma concentration time profile adequately. In case of mild hepatic impairment, plasma protein scale factor was reduced to 0.85 to account for the higher C_max_ seen in these patients. For the moderate hepatic impairment group this scale factor was set to 1.30, which resulted in an increased volume of distribution and consequently in a lower C_max_. The higher fu_p_ assumed for the moderate liver impaired population led to an increased clearance, resulting in lower plasma concentration levels throughout the curve and a slightly lower increase in plasma exposure compared to the predicted AUC_last_ for the mild liver impaired population.

## 4. Discussion

CAB is a new drug, which could be used in other therapeutic areas, besides RCC and HCC, as it is investigated in numerous clinical trials. As an example, the safety and efficacy of CAB in advanced adrenocortical carcinoma is currently evaluated [49]. Despite the increasingly widespread use, not all pharmacokinetic properties of the substance have been studied in all detail. In the presented work, a whole-body PBPK model for CAB has been established, which was less used as a precision tool but rather as a tool to serve as a further step to gain insights into the PK properties of the substance. The developed model successfully describes and predicts observed plasma concentration time profiles of different CAB formulations (solution, capsule, and tablet) in a wide dose range (20–140 mg) after single-dose administration in humans and for ig (15–30 mg/kg) and iv (5–10 mg/kg) administration in rats. The inspection of rat plasma concentration time profiles after ig administration hypothesized nonlinear PK (Figure S3), as for the lower dose, significantly higher plasma concentrations were observed if plotted dose normalized. This is not the case after iv administration, which led to the conclusion that the nonlinear PK seen in the ig profiles might be associated with drug absorption processes. The optimization of relevant parameters showed that especially in the large intestine but also in the ileum the intestinal solubility was overestimated and reduced accordingly. Inspection of the human plasma concentration time profiles (Figures S5 and S6) revealed a second peak approximately 24 h after CAB administration. In addition, according to a study by Lacy et al., relatively high ^14^C-CAB levels in faeces one week post-dose can be observed. Both phenomena could be justified as a consequence of an EHC of CAB [13,50]. Seventeen metabolites of CAB were identified, of which EXEL-1646 is the only human metabolite that could be back transformed to the parent compound (see ESM Figure S1) [13]. However, it has been shown in vitro that EXEL-1646 will only be transformed into its nonconjugated monohydroxy precursor [13]. In contrast, rat plasma concentration time profiles do not feature the phenomenon of a second or multiple peaks post-dose (Figure S3), which might be due to the missing gallbladder in rats and in consequence due to the un-incisive EHC. In the CAB assessment report, an EHC of the substance is assumed as most possible cause for the characteristic PK profile of CAB in humans, which served as a starting point for model development [51,52]. However, alternative processes might also explain multiple peaks in plasma concentration time profile and a long terminal half-life combined with a steeper decrease in the plasma concentration at the beginning. For example, absorption processes in deeper intestine sections, like in the upper or lower ileum, could cause a delayed absorption. To verify this possibility, the fractions absorbed in different intestine sections were simulated and confirmed a lower fraction absorbed for the capsule formulation compared to the tablet formulation and solution. This is in accordance with the findings by Lacy et al., who stated that the mean AUC_0–inf_ values for HVs administered a 140 mg CAB capsule dose or tablet dose were 74–93% and 97%, respectively, of the corresponding value in the mass balance study where CAB was administered as a solution [12]. Besides a lower total fraction absorbed, a delay in the absorption process could be observed in the simulations with lower fractions absorbed in the first 24 h for capsules compared to tablets and to the solution. Nevertheless, the delayed absorption alone could not account for the long terminal half-life. Therefore, the EHC process was implemented into the model, which notably improved its performance. Comparisons of simulations with and without the EHC process highlighted the strong impact of the process on CAB plasma exposure. After all, a combination of delayed intestinal absorption and EHC led to the best description of CAB plasma concentration time profiles.

The final PBPK model also included an active MRP2 transport process and CYP3A4 metabolism, two main features characterizing CAB PK and representing possible sources of DDI and resistance [18,41,53]. CAB is mainly used in advanced disease states, e.g., in hepatocellular carcinoma, where co-administration of several additional drugs is not uncommon and several authors [9,54] discussed this issue. Interactions with strong CYP3A4 inducers (e.g., Rifampin) or inhibitors (e.g., Ketoconazole) can lead to decreased or increased CAB plasma concentrations, which may require closer monitoring and dose adjustment [41]. The developed model was used to simulate DDI with RIF and confirmed a decrease of 77% in plasma exposure, when RIF is co-administered with CAB. The model adequately describes the CAB RIF DDI plasma concentration time profile and is in good agreement with the observed data. The correct prediction of the impact of the perpetrator drug indicates the correct implementation of the relevant elimination pathways of the victim drug and the right amount of drug eliminated via that pathway [55]. After a single dose administration, it was not possible to match AUC_last_ values of the control data by only increasing the amount of the single dose, as in that case, C_max_ will also strongly increase, which could have an impact on safety. However, CAB accumulates five-fold by day 15 following daily dosing based on AUC, which should be considered in the treatment of these patients. As no clinical data after multiple-dose administration were available, predicted C_ss_ after multiple dosing for the 60 mg tablet was compared to the C_ss_ published by Castellano et al. [56], to evaluate the ability of the model to simulate multiple dosing correctly. A good agreement between the predicted and published value was found (1197 ng/mL vs. 1123 ng/mL). Steady state simulations for CAB co-administered with RIF indicate that a dose increase by 20 mg (tablet) and 40 mg (capsule), respectively might not be sufficient to achieve the corresponding CAB plasma levels observed without perpetrator. Hence, the model presented in this work cannot confirm, that the dose increase recommended by FDA will lead to equivalent C_ss_ (Figure S10). It may be for this reason that EMA advises against using a strong CYP3A4 inducer, when patients are treated with CAB to avoid subtherapeutic plasma concentrations. However, there may be situations where it is clinically necessary to co-administer both substances and slightly lower CAB plasma levels are still sufficient. To counteract CAB C_ss_ that are far below the desired threshold, it might be valuable to conduct therapeutic drug monitoring in patients treated concomitantly with CAB and a strong CYP3A4 inducer like RIF.

Limitations, which could reduce the applicability of the model, should be highlighted. No human iv data were available and the human PBPK model of CAB was developed based on peroral data only. This is generally possible [28] but leads to a number of uncertainties and inaccuracies. A state-of-the-art approach is to establish an iv model first and to describe distribution and clearance processes independently from drug absorption processes. In a second step, the model for p.o. administration can be established based on the iv model and parameters that influence absorption can be estimated with less uncertainty. To overcome this issue, a cross-species sequential approach using rat iv data was pursued. Based on these simulations for the rat, relevant physicochemical properties were evaluated to describe drug distribution. However, clearance processes and especially the EHC process could not be transferred from rat to human, as information on rat clearance is limited, the rat has no gallbladder, and metabolism processes are fundamentally different between rats and human, as stated by Lacy et al. [13]. One shortcoming is that the original study data were not available. Therefore, for model development only mean plasma concentrations without observed standard deviations extracted from the literature could be used, which made it impossible to assess interindividual variability. Lacy et al. reported multiple peaks in the plasma concentration time profiles after a single oral dose of CAB [12]. However, in the mean plasma concentration time profiles these multiple peaks cannot be observed, but they might be present in individual plasma concentration time profiles. In the case of EHC, one might expect peaks earlier than 24 h after administration, matching with the food intake of the volunteers, but only two plasma concentration time profiles show small peaks already approximately nine hours after CAB administration. However, as EHC is characterized by high interindividual variability [57], mean data might not accurately represent the individual course. Model inaccuracy may also result from the periodicity of the complex EHC process, dependent on digestion with high interindividual variability, e.g., on bile accumulation, concentration, and release into the duodenum. The high variability of the EHC process was also mentioned in a published PBPK model for Sorafenib, another TKI approved for HCC, RCC and MTC. The work of Abbiati et al. highlights the EHC, causing the characteristic double peak in the plasma concentration time curves of Sorafenib [50]. In contrast to the CAB model, the model of Abbiati et al. was developed based on mice data and is restricted to the first EHC contribution due to limited available data regarding this process.

Van Erp et al. point out that for TKI “most of the available PK information is based on information obtained from in vitro experiments, animal studies, DDI studies and mass balance studies in HVs with a single dose of the aimed TKI. However, it is difficult to translate the results of these studies to the clinical oncology practice where these drugs are administered on a daily basis […]” [58]. The CAB PBPK model was also developed based on HVs but now gathers all information and combines it with relevant physiological and anatomical processes, which are integrated in the PK-Sim^®^ software.

With that, the model could be extended to simulate the plasma exposure in patients with mild and moderate liver impairment. Liver diseases can affect several morphological and physiological processes and may have an impact on enterohepatic circulation and biliary excretion. A decreased uptake into hepatocytes, an altered metabolism, and distribution within the hepatocytes as well as reduced transfer mechanism into bile is conceivable. Especially cholestasis can have a significant influence on hepatobiliary excretion, either through biliary obstruction (e.g., caused by duct stones or pancreatic carcinoma) or transporter defects and downregulation, respectively (e.g., strong downregulation of canalicular MRP2). However, patients in the study of the effect of hepatic impairment on CAB PK were not affected by cholestasis but by liver cirrhosis only [37]. According to Roberts et al. [57], liver cirrhosis rather affects sinusoidal membrane transport systems than canalicular bile transport systems. Therefore, the focus was placed on changes in hepatic blood flow, in cellular enzymatic function, and in altered plasma protein binding to simulate patients suffering from mild and moderate liver impairment. According to Nguyen et al. [37], plasma exposure was increased by 81% and 63% in patients with mild, respectively moderate liver impairment, compared to healthy subjects. Simulation using the herein presented model yielded a slightly lower increase in plasma exposure by 64% and 50% for mild and moderate liver impairment but it meets the observed tendency, and it must be noted that hepatic dysfunction varies widely between patients with the same Child-Pugh score. A lower increase in plasma exposure for moderate disease compared to subjects with mild hepatic impairment and a generally lower C_max_ for those patients was justified according to Nguyen et al. by a higher fu_p_ and a higher interindividual variability. The model was able to describe C_max_ and AUC_last_ in patients with mild and moderate hepatic impairment correctly and can be expanded for further investigations, for example, in patients with Child-Pugh class C, as there is a knowledge gap for such patients.

It must be considered that the simulated populations with liver disease contained only male individuals, as the underlying study was conducted exclusively in male subjects. The transferability to females is, therefore, limited. In addition, patients of the underlying study where considerably older than the individual used for model development (37 years vs. 54 to 58 years). The model was also developed based on an individual with lower body weight (73.96 kg) and BMI, respectively compared to the participants from the hepatic impairment study (mean weight control group: 86.00 kg, mean weight mild hepatic impairment group: 92.40 kg, mean weight moderate hepatic impairment group: 88.50 kg). BMI can have an impact on oral plasma clearance. It was shown that with higher BMI, the CAB apparent clearance decreases [59], which could have also influenced the simulated plasma exposure. A reduction in plasma proteins can be associated with different disease states and results in altered fu_p_ [60,61]. Slight changes in fu_p_ tend to have a higher impact on compounds with excessive protein binding such as CAB because the observed percent change would be large. The presented model revealed a considerable influence of the CAB fu_p_ on plasma exposure and C_max._ In relevant patients, suffering from cirrhosis, it might be valuable to measure this parameter to predict CAB PK more precisely.

CAB is used in cancer patients and several other alterations regarding physiology are possible, both because of the disease itself or due to co-medication, and could be adapted in the developed model as it was done for liver cirrhosis [62]. EHC process, CYP3A4 and MRP2 expression and activity can be modified, and the model can, therefore, serve as a starting point to transfer the results to populations of interest. Comorbidities like cholecystitis, cholestasis or cholelithiasis but also diabetes mellitus and renal diseases could influence enterohepatic circulation directly or indirectly and might be subject of future questions [57]. As already stated, CAB plasma levels could be altered through its affinity to MRP2 transporter and it is conceivable to investigate situations, which could lead to alterations in MRP2 expression, like cholestatic liver diseases [63,64,65], based on the presented model. Concomitant use of drugs that inhibit MRP2 transport could result in higher plasma concentrations whereas an overexpression of MRP2, e.g., in specific tumours or due to a concomitant use of pregnane X receptor ligand (e.g., Phenobarbital) might lead to the opposite [64,66,67,68] effect. Both scenarios are more than unfavourable for the patients, as they either can suffer from side effects or less efficacy. As DDI based on the CAB CYP3A4 metabolism are very important, with more data becoming available in the future, the model might be used as a basis for further investigations regarding relevant DDI and the associated dose adaptations.

## 5. Conclusions

A whole body PBPK model for CAB has been built in PK-Sim^®^ including key processes like CYP3A4 metabolism, MRP2 transport and EHC which may influence pharmacokinetic properties of the drug. It comprises in vitro, in vivo, and in silico information combined with available CAB plasma concentrations. EHC was found to be the most plausible cause for the characteristic PK profile. The final model is characterized by good precision and low bias. The model was expanded to simulate DDI with RIF and CAB plasma exposure in patients with mild and moderate liver impairment.

PBPK modeling is a convenient tool to mechanistically explore the PK behavior of a drug and the model presented in this work can contribute to gain further insights into CAB absorption, distribution, metabolism, and excretion. As a comprehensive software tool was used in this work, it will be convenient to reuse the PBPK model for other scenarios also by non-modeling experts. By that, it serves as a starting point for further studies in specific populations, disease states or with respect to drug interactions (e.g., via CYP3A4). So far, clinical study data are limited, especially with regard to plasma exposure after multiple dosing, but they might be available in the near future with more studies being conducted. Future PBPK models could be developed by implementing data from cancer patients or from patients taking relevant co-medication.

## Figures and Tables

**Table 1:** Summary of the CAB parameters used in the human PBPK model.

Parameter	Unit	Value Used in PBPK Model	Literature Value [Reference]	Description
MW	[g/mol]	501.50	501.50 [44]	Molecular weight
*p*K_a_ [base]		6.32	6.32 [45]	Acid dissociation constant
fu_p_		0.24	0.24 [13]	Fraction unbound in plasma
logP		4.40 ^a^	5.15 [45]	Lipophilicity
Solubility (pH 6.5)	[10^−3^ mg/mL]	7.72 ^a^	0.00 [44]	Solubility
K_M_ CYP3A4	[µmol/L]	0.97 ^a^	21.32 [43]	Michaelis-Menten constant CYP3A4
k_cat_ CYP3A4	[1/min]	0.67	0.66 [43]	Katalytic rate constant CYP3A4
K_M_ MRP2	[µmol/L]	10 ^a^	--	Michaelis-Menten constant MRP2
k_cat_ MRP2	[1/min]	2111.11 ^a^	--	Transport rate constant MRP2
Reference concentration MRP2	[µmol protein/l in the tissue of highest expression]	0.09	0.06 [46]	Liver reference concentration
Partition coefficients		Rodgers and Rowland	[47,48]	Calculation method cell to plasma coefficients
Cellular permeabilities		PKSim^®^ Standard	[29]	Calculation method permeation across cell membranes
Transcellular intestinal permeability	[10^−4^ cm/min]	1.70 ^a^	--	Intestinal permeability via transcellular route
Tablet Weibull time	[min]	36.00 ^a^		Dissolution time (50% dissolved) fasted state
Tablet Weibull shape		1.29 ^a^		Shape parameter of Weibull function
Capsule Weibull time	[min]	45.00		Dissolution time (50% dissolved) fasted state
Capsule Weibull shape		5.00		Shape parameter of Weibull function
Emptying half-time	[min]	41.44	69.98 [33]	Half-time for gallbladder emptying (exponential release)
EHC continuous fraction		0.1		Fraction of biliary secreted compound continuously entering duodenum
Gallbladder ejection fraction		0.45		Fraction discretely ejected into the duodenum
Refilling time	[min]	241.00	419 [33]	Time to complete gallbladder refilling

^a^ Model parameters have been estimated through parameter optimization based on the plasma drug concentrations, EHC: Enterohepatic circulation, -- Value not available.

**Table 2:** Comparison of AUC_last_ and C_max_ for the administration of CAB, given either alone or in combination with its perpetrator RIF.

PK Parameter	CAB with RIF	CAB without RIF	Ratio
AUC_last_	13,624.61	59,738.08	0.23
C_max_	480.46	551.17	0.87

**Table 3:** Physiological changes of parameters associated with hepatic impairment.

Parameter	Control Group	Mild Hepatic Impairment	Moderate Hepatic Impairment
Hematocrit value ^b^	0.47 ^a^	0.39 ^b^	0.37 ^b^
Blood flow ^b^			
portal	1.00	0.40	0.36
renal	1.00	0.88	0.65
hepatic arterial	1.00	1.30	2.30
other organs	1.00	1.75	2.25
Liver volume fraction^b^	1.00	0.81	0.65
CYP3A4 activity	1.00	1.00	0.40
Plasma protein scale factor	1.00 ^a^	0.85	1.25

^a^ value based on PKSim^®^, ^b^ values according to Edginton et al. [38].

## Data Availability

All modeling files including utilized clinical study data can be found here: https://github.com/Open-Systems-Pharmacology (accessed on 30 April 2021).

## Supplementary Materials

The following are available online at https://www.mdpi.com/article/10.3390/pharmaceutics13060778/s1, Electronic Supplementary Materials: Additional information on model development and evaluation including Figures S1–S11 and Tables S1–S7.

## References

[1] Kurzrock R., Sherman S.I., Ball D.W., Forastiere A.A., Cohen R.B., Mehra R., Pfister D.G., Cohen E.E., Janisch L., Nauling F. (2011). Activity of XL184 (Cabozantinib), an oral tyrosine kinase inhibitor, in patients with medullary thyroid cancer. J. Clin. Oncol..

[2] Markowitz J.N., Fancher K.M. (2018). Cabozantinib: A multitargeted oral tyrosine kinase inhibitor. Pharmacotherapy.

[3] Neul C., Schaeffeler E., Sparreboom A., Laufer S., Schwab M., Nies A.T. (2016). Impact of Membrane Drug Transporters on Resistance to Small-Molecule Tyrosine Kinase Inhibitors. Trends Pharm. Sci..

[4] Grüllich C. (2014). Cabozantinib: A MET, RET, and VEGFR2 tyrosine kinase inhibitor. Small Molecules in Oncology.

[5] Fallahi P., Ferrari S.M., Di Bari F., Materazzi G., Benvenga S., Miccoli P., Antonelli A. (2015). Cabozantinib in thyroid cancer. Recent Pat. Anticancer Drug Discov..

[6] Singh H., Brave M., Beaver J.A., Cheng J., Tang S., Zahalka E., Palmby T.R., Venugopal R., Song P., Liu Q. (2017). US Food and Drug Administration approval: Cabozantinib for the treatment of advanced renal cell carcinoma. Clin. Cancer Res..

[7] Tannir N.M., Schwab G., Grünwald V. (2017). Cabozantinib: An active novel multikinase inhibitor in renal cell carcinoma. Curr. Oncol. Rep..

[8] Abou-Alfa G.K., Meyer T., Cheng A.-L., El-Khoueiry A.B., Rimassa L., Ryoo B.-Y., Cicin I., Merle P., Chen Y., Park J.-W. (2018). Cabozantinib in Patients with Advanced and Progressing Hepatocellular Carcinoma. N. Engl. J. Med..

[9] Deeks E.D. (2019). Cabozantinib: A Review in Advanced Hepatocellular Carcinoma. Target. Oncol..

[10] Yakes F.M., Chen J., Tan J., Yamaguchi K., Shi Y., Yu P., Qian F., Chu F., Bentzien F., Cancilla B. (2011). Cabozantinib (XL184), a novel MET and VEGFR2 inhibitor, simultaneously suppresses metastasis, angiogenesis, and tumor growth. Mol. Cancer Ther..

[11] U.S. National Library of Medicine ClinicalTrials.gov. Search Term: “Cabozantinib”. https://clinicaltrials.gov/ct2/results?cond=&term=Cabozantinib&cntry=&state=&city=&dist=.

[12] Lacy S.A., Miles D.R., Nguyen L.T. (2017). Clinical Pharmacokinetics and Pharmacodynamics of Cabozantinib. Clin. Pharm..

[13] Lacy S., Hsu B., Miles D., Aftab D., Wang R., Nguyen L. (2015). Metabolism and Disposition of Cabozantinib in Healthy Male Volunteers and Pharmacologic Characterization of Its Major Metabolites. Drug Metab. Dispos..

[14] Ren L.J., Wu H.J., Sun L.H., Xu X., Mo L.Y., Zhang L., Zhang J.Y., Wu C.Y. (2018). A sensitive LC-MS/MS method for simultaneous determination of cabozantinib and its metabolite cabozantinib N-oxide in rat plasma and its application in a pharmacokinetic study. Biomed. Chromatogr..

[15] Lehr T., Staab A., Tillmann C., Trommeshauser D., Schaefer H.-G., Kloft C. (2009). A quantitative enterohepatic circulation model. Clin. Pharm..

[16] Metsugi Y., Miyaji Y., Ogawara K.-I., Higaki K., Kimura T. (2008). Appearance of double peaks in plasma concentration–time profile after oral administration depends on gastric emptying profile and weight function. Pharm. Res..

[17] Suttle A.B., Pollack G.M., Brouwer K.L. (1992). Use of a pharmacokinetic model incorporating discontinuous gastrointestinal absorption to examine the occurrence of double peaks in oral concentration–time profiles. Pharm. Res..

[18] Nguyen L., Holland J., Miles D., Engel C., Benrimoh N., O’Reilly T., Lacy S. (2015). Pharmacokinetic (PK) drug interaction studies of cabozantinib: Effect of CYP3A inducer rifampin and inhibitor ketoconazole on cabozantinib plasma PK and effect of cabozantinib on CYP2C8 probe substrate rosiglitazone plasma PK. J. Clin. Pharm..

[19] European Medicines Agency Cabometyx Summary of Product Characteristics. https://www.ema.europa.eu/en/documents/product-information/cabometyx-epar-product-information_en.pdf.

[20] U.S. Food and Drug Administration (2018). Physiologically Based Pharmacokinetic Analyses–Format and Content–Guidance for Industry. https://www.fda.gov/regulatory-information/search-fda-guidance-documents/physiologically-based-pharmacokinetic-analyses-format-and-content-guidance-industry.

[21] Peters S.A. (2012). Physiologically-Based Pharmacokinetic (PBPK) Modeling and Simulations: Principles, Methods, and Applications in the Pharmaceutical Industry.

[22] Jones H., Rowland-Yeo K. (2013). Basic concepts in physiologically based pharmacokinetic modeling in drug discovery and development. CPT Pharmacomet. Syst. Pharmacol..

[23] Schmitt W., Willmann S. (2004). Physiology-based pharmacokinetic modeling: Ready to be used. Drug Discov. Today Technol..

[24] Jones H.M., Parrott N., Jorga K., Lavé T. (2006). A novel strategy for physiologically based predictions of human pharmacokinetics. Clin. Pharm..

[25] Open Systems Pharmacology PK-Sim®. Version 7.4.0. https://github.com/Open-Systems-Pharmacology/Suite/releases/tag/7.4.0.

[26] Eissing T., Kuepfer L., Becker C., Block M., Coboeken K., Gaub T., Goerlitz L., Jaeger J., Loosen R., Ludewig B. (2011). A computational systems biology software platform for multiscale modeling and simulation: Integrating whole-body physiology, disease biology, and molecular reaction networks. Front. Physiol..

[27] Willmann S., Lippert J., Sevestre M., Solodenko J., Fois F., Schmitt W. (2003). PK-Sim^®^: A physiologically based pharmacokinetic ‘whole-body’ model. Biosilico.

[28] Kuepfer L., Niederalt C., Wendl T., Schlender J.F., Willmann S., Lippert J., Block M., Eissing T., Teutonico D. (2016). Applied Concepts in PBPK Modeling: How to Build a PBPK/PD Model. CPT Pharmacomet. Syst. Pharmacol..

[29] Open Systems Pharmacology PK-Sim® Software Manual. https://docs.open-systems-pharmacology.org/.

[30] R Core Team (2020). R: A Language and Environment for Statistical Computing.

[31] Brightman F.A., Leahy D.E., Searle G.E., Thomas S. (2006). Application of a generic physiologically based pharmacokinetic model to the estimation of xenobiotic levels in rat plasma. Drug Metab. Dispos..

[32] Jedlitschky G., Hoffmann U., Kroemer H.K. (2006). Structure and function of the MRP2 (ABCC2) protein and its role in drug disposition. Expert Opin. Drug Metab. Toxicol..

[33] Baier V., Cordes H., Thiel C., Castell J.V., Neumann U.P., Blank L.M., Kuepfer L. (2019). A Physiology-Based Model of Human Bile Acid Metabolism for Predicting Bile Acid Tissue Levels After Drug Administration in Healthy Subjects and BRIC Type 2 Patients. Front. Physiol..

[34] European Medicines Agency (2018). Guideline on the Reporting of Physiologically Based Pharmacokinetic (PBPK) Modelling and Simulation. https://www.ema.europa.eu/en/reporting-physiologically-based-pharmacokinetic-pbpk-modelling-simulation.

[35] Kuemmel C., Yang Y., Zhang X., Florian J., Zhu H., Tegenge M., Huang S.M., Wang Y., Morrison T., Zineh I. (2020). Consideration of a credibility assessment framework in model-informed drug development: Potential application to physiologically-based pharmacokinetic modeling and simulation. CPT Pharmacomet. Syst. Pharmacol..

[36] Hanke N., Frechen S., Moj D., Britz H., Eissing T., Wendl T., Lehr T. (2018). PBPK models for CYP3A4 and P-gp DDI prediction: A modeling network of rifampicin, itraconazole, clarithromycin, midazolam, alfentanil, and digoxin. CPT Pharmacomet. Syst. Pharmacol..

[37] Nguyen L., Holland J., Ramies D., Mamelok R., Benrimoh N., Ciric S., Marbury T., Preston R.A., Heuman D.M., Gavis E. (2016). Effect of Renal and Hepatic Impairment on the Pharmacokinetics of Cabozantinib. J. Clin. Pharm..

[38] Edginton A.N., Willmann S. (2008). Physiology-based simulations of a pathological condition. Clin. Pharm..

[39] Johnson T.N., Boussery K., Rowland-Yeo K., Tucker G.T., Rostami-Hodjegan A. (2010). A semi-mechanistic model to predict the effects of liver cirrhosis on drug clearance. Clin. Pharm..

[40] George J., Murray M., Byth K., Farrell G.C. (1995). Differential alterations of cytochrome P450 proteins in livers from patients with severe chronic liver disease. Hepatology.

[41] Martignoni M., Groothuis G.M., de Kanter R. (2006). Species differences between mouse, rat, dog, monkey and human CYP-mediated drug metabolism, inhibition and induction. Expert Opin. Drug Metab. Toxicol..

[42] Colclough N., Ruston L., Wood J.M., MacFaul P.A. (2014). Species differences in drug plasma protein binding. MedChemComm.

[43] Lin Q.M., Li Y.H., Lu X.R., Wang R., Pang N.H., Xu R.A., Cai J.P., Hu G.X. (2019). Characterization of Genetic Variation in CYP3A4 on the Metabolism of Cabozantinib in Vitro. Chem. Res. Toxicol..

[44] U.S. Food and Drug Administration (2015). CABOMETYX (cabozantinib) Tablets. Chemistry Review(s). https://www.accessdata.fda.gov/drugsatfda_docs/nda/2016/208692Orig1s000ChemR.pdf.

[45] Ipsen Biopharmaceuticals Canada Inc. (2019). CABOMETYX Product Monograph. https://ipsen.com/websites/IPSENCOM-PROD/wp-content/uploads/sites/18/2019/11/21094828/Cabometyx-PM-EN-07Nov2019.pdf.

[46] Deo A.K., Prasad B., Balogh L., Lai Y., Unadkat J.D. (2012). Interindividual variability in hepatic expression of the multidrug resistance-associated protein 2 (MRP2/ABCC2): Quantification by liquid chromatography/tandem mass spectrometry. Drug Metab. Dispos..

[47] Rodgers T., Leahy D., Rowland M. (2005). Physiologically based pharmacokinetic modeling 1: Predicting the tissue distribution of moderate-to-strong bases. J. Pharm. Sci..

[48] Rodgers T., Rowland M. (2006). Physiologically based pharmacokinetic modelling 2: Predicting the tissue distribution of acids, very weak bases, neutrals and zwitterions. J. Pharm. Sci..

[49] Kroiss M., Megerle F., Kurlbaum M., Zimmermann S., Wendler J., Jimenez C., Lapa C., Quinkler M., Scherf-Clavel O., Habra M.A. (2020). Objective response and prolonged disease control of advanced adrenocortical carcinoma with cabozantinib. J. Clin. Endocrinol. Metab..

[50] Abbiati R.A., Manca D. (2017). Enterohepatic Circulation Effect in Physiologically Based Pharmacokinetic Models: The Sorafenib Case. Ind. Eng. Chem. Res..

[51] European Medicines Agency (2013). Assessment Report Cometriq. https://www.ema.europa.eu/en/documents/assessment-report/cometriq-epar-public-assessment-report_en.pdf.

[52] European Medicines Agency (2016). CHMP Assessment Report Cabometyx. https://www.ema.europa.eu/en/documents/assessment-report/cabometyx-epar-public-assessment-report_en.pdf.

[53] Schinkel A.H., Jonker J.W. (2012). Mammalian drug efflux transporters of the ATP binding cassette (ABC) family: An overview. Adv. Drug Deliv. Rev..

[54] D’Angelo A., Sobhani N., Bagby S., Casadei-Gardini A., Roviello G. (2020). Cabozantinib as a second-line treatment option in hepatocellular carcinoma. Expert Rev. Clin. Pharm..

[55] Fuhr L.M., Marok F.Z., Hanke N., Selzer D., Lehr T. (2021). Pharmacokinetics of the CYP3A4 and CYP2B6 Inducer Carbamazepine and Its Drug–Drug Interaction Potential: A Physiologically Based Pharmacokinetic Modeling Approach. Pharmaceutics.

[56] Castellano D., Maroto J.P., Benzaghou F., Taguieva N., Nguyen L., Clary D.O., Jonasch E. (2020). Exposure-response modeling of cabozantinib in patients with renal cell carcinoma: Implications for patient care. Cancer Treat. Rev..

[57] Roberts M.S., Magnusson B.M., Burczynski F.J., Weiss M. (2002). Enterohepatic circulation. Clin. Pharm..

[58] van Erp N.P., Gelderblom H., Guchelaar H.-J. (2009). Clinical pharmacokinetics of tyrosine kinase inhibitors. Cancer Treat. Rev..

[59] Miles D., Jumbe N.L., Lacy S., Nguyen L. (2016). Population pharmacokinetic model of cabozantinib in patients with medullary thyroid carcinoma and its application to an exposure-response analysis. Clin. Pharmacokinet..

[60] Spinella R., Sawhney R., Jalan R. (2016). Albumin in chronic liver disease: Structure, functions and therapeutic implications. Hepatol. Int..

[61] Carvalho J.R., Machado M.V. (2018). New insights about albumin and liver disease. Ann. Hepatol..

[62] Cheeti S., Budha N.R., Rajan S., Dresser M.J., Jin J.Y. (2013). A physiologically based pharmacokinetic (PBPK) approach to evaluate pharmacokinetics in patients with cancer. Biopharm. Drug Dispos..

[63] Oswald M., Kullak-Ublick G.A., Paumgartner G., Beuers U. (2001). Expression of hepatic transporters OATP-C and MRP2 in primary sclerosing cholangitis. Liver.

[64] Pauli-Magnus C., Meier P.J. (2006). Hepatobiliary transporters and drug-induced cholestasis. Hepatology.

[65] Borst P., Zelcer N., Van De Wetering K. (2006). MRP2 and 3 in health and disease. Cancer Lett..

[66] Schrenk D., Baus P.R., Ermel N., Klein C., Vorderstemann B., Kauffmann H.-M. (2001). Up-regulation of transporters of the MRP family by drugs and toxins. Toxicol. Lett..

[67] Kullak-Ublick G.A., Stieger B., Meier P.J. (2004). Enterohepatic bile salt transporters in normal physiology and liver disease. Gastroenterology.

[68] Mottino A.D., Catania V.A. (2008). Hepatic drug transporters and nuclear receptors: Regulation by therapeutic agents. World J. Gastroenterol. WJG.

