# Supplementary material for: Physiologically Based Pharmacokinetic Modelling of Cabozantinib to Simulate Enterohepatic Recirculation, Drug–Drug Interaction with Rifampin and Liver Impairment
Source: Pharmaceutics. 2021 May 22;13(6):778. doi: 10.3390/pharmaceutics13060778 (PMC8224782; doi:10.3390/pharmaceutics13060778)
Supplement: Supplementary file 1 [file pharmaceutics-13-00778-s001.zip › pharmaceutics-1224709-SI.pdf]

pharmaceutics

Supplementary Materials: Physiologically Based Pharmacokinetic Modelling of Cabozantinib to Simulate Enterohepatic Recirculation, Drug–Drug Interaction with Rifampin and Liver Impairment

Bettina Gerne and Oliver Scherf-Clavel

1. Cabozantinib PBPK Model Development

In this study, a physiologically based pharmacokinetic (PBPK) model for the oral tyrosine kinase inhibitor cabozantinib (CAB) was developed. The substance is extensively metabolized by CYP3A4 and four major CAB metabolites can be found in plasma, namely Exel 5366 (Amide Cleavage Product), Exel 1644 (6-Desmethyl Amide Cleavage Product Sulfate), Exel 1646 (Monohydroxy Sulfate) and Exel 5162 (N-Oxide) (Figure S1). The relevance of membrane transporters for in vivo drug disposition is still unclear [1]. CAB was tested as a substrate for various transporter and was found to be a substrate of MRP2 only [2]. Being a Biopharmaceutics Classification System Class II (BCS II) compound, CAB is characterized by low water solubility and a high cell permeability [3]. As a weak base, only a very small proportion of CAB is charged at the physiological pH of 7.4 and therefore negligible affinity can be expected between CAB and organic anion transporters. Hence, passive diffusion is being considered as the only way for CAB to cross biomembranes in the present model except for the MRP2 mediated active secretion into bile. The PBPK model for CAB was developed to test the hypothesis of EHC and to confirm factors that may influence CAB PK behaviour. The concomitant administration of the strong CYP3A4 inducer Rifampin (RIF) and the influence of liver impairment was investigated regarding changes in plasma concentration-time profiles and exposure. An intensive literature search was conducted for drug-specific model input parameters and 14 plasma concentration time profiles from seven human clinical studies were digitized, divided into a training ($n = 6$) or a test ($n = 4$) dataset and used for model development and evaluation or were used to simulate DDI and liver impairment (Table S1). The model development process was supplemented with intravenous (i.v.) (5 mg/kg, 10mg/kg) data from rats, found in a published work by Wang et al. [4]. All sampling time points for rat and human blood samples [3mL] in each study are given in Table S2. The allocation of human plasma profiles to the training dataset has been done in such a way that i) the broadest possible dose range was covered (20 mg to 140 mg) and ii) both formulations were included (capsule and tablet). For model input parameters which could not be found in literature the parameter identification function in PKSim® was used and model simulations of all training datasets were fitted to the observed data with the integrated Monte Carlo algorithm. The workflow of the CAB PBPK model development from rats to the final CAB PBPK model in humans is shown in Figure S2.

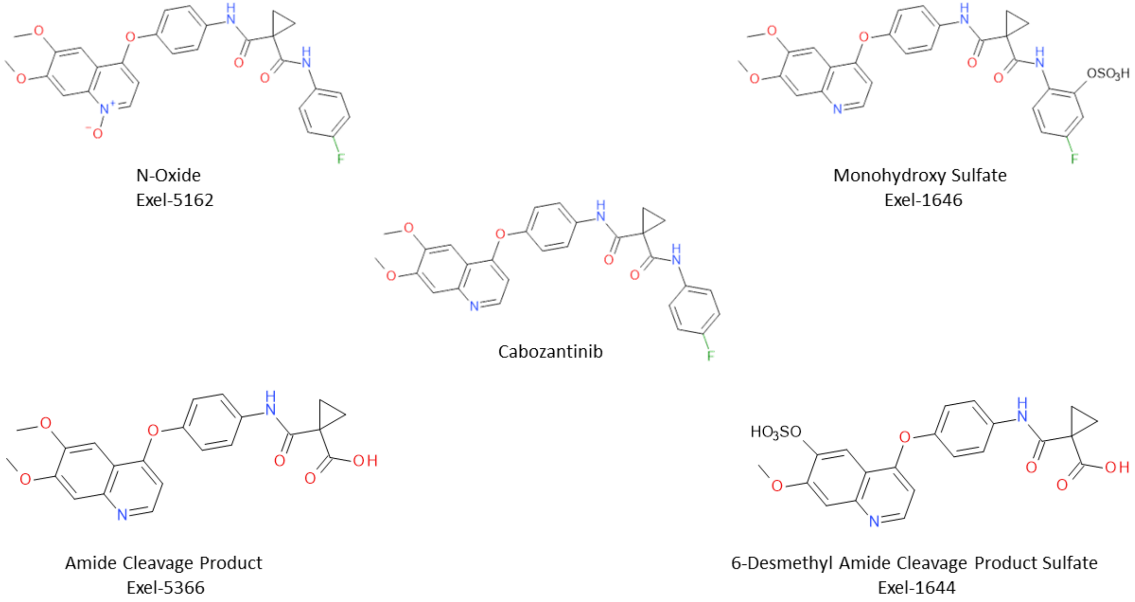

Figure S1. Cabozantinib and its four major plasma metabolites, Exel 5366, Exel 1644, Exel 1646 and Exel 5162.

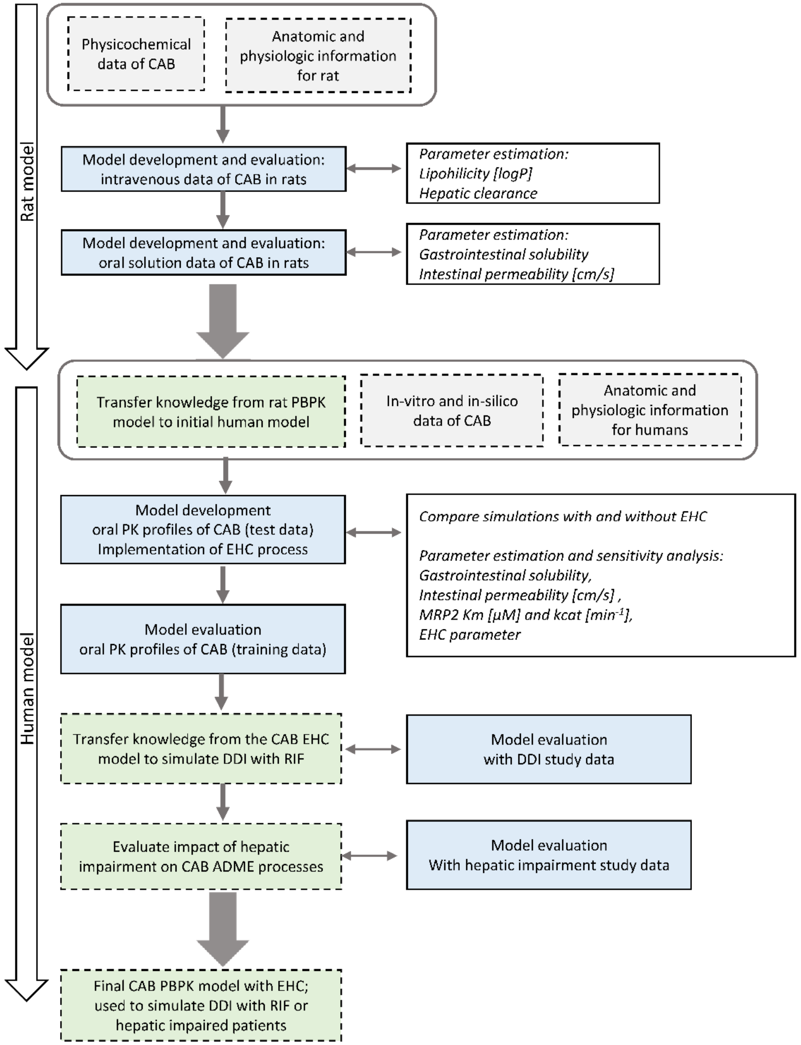

Figure S2. Workflow of the CAB PBPK model development from rats to the final model in humans, which contains enterohepatic circulation process and is capable to model DDI with Rifampin or plasma concentration–time profiles in hepatic impaired patients. CAB: cabozantinib; DDI: drug-drug interaction; EHC: enterohepatic circulation; RIF: rifampin.

Table S1. Experimental datasets used for development and evaluation of the base CAB PBPK model, for DDI and hepatic impairment simulations.

Study	Dose [mg]	Treatment	n	Men [%]	Age [yrs]	Weight [kg]	Height [cm]	Dataset	References
PK in rats, Wang et al.	5 mg/kg	iv, SD	8	n.r.	n.r.	n.r.	n.r.	training	[4]
PK in rats, Wang et al.	10 mg/kg	iv, SD	8	n.r.	n.r.	n.r.	n.r.	training	[4]
PK in rats, Wang et al.	15 mg/kg	ig, SD	8	n.r.	n.r.	n.r.	n.r.	training	[4]
PK in rats, Wang et al.	30 mg/kg	ig, SD	8	n.r	n.r.	n.r	n.r.	training	[4]
Mass Balance Study, Lacy et al.	140	po, solution, SD	8	100	n.r. (19-55)	n.r.	n.r.	training	[2]
Phase I PK, Nguyen et al.	20	po, tab, SD	21	52	41 (24-54)	79 (61-112)	168 (151-184)	training	[5]
Phase I PK, Nguyen et al.	40	po, tab, SD	21	52	35 (19-49)	76 (60-97)	166 (152-184)	test	[5]
Phase I PK, Nguyen et al.	60	po, tab, SD	21	52	35 (21-49)	76 (59-93)	165 (145-182)	test	[5]
Phase I BE, Nguyen et al.	140	po, tab, SD	77	42	39 (18-55)	72 (46-108)	164 (146-189)	training	[5]
Phase I BE, Nguyen et al.	140	po, cap, SD	77	42	39 (18-55)	72 (46-108)	164 (146-189)	training	[5]
DDI Study 1, Nguyen et al.	140	po, cap, SD, with RIF	28	57	35 (22-49)	77 (57-111)	n.r.	DDI	[6], Study 1
DDI Study 1, Nguyen et al.	140	po, cap, SD, w/o RIF	28	57	35 (22-49)	77 (57-111)	n.r.	test	[6], Study 1
DDI Study 2, Nguyen et al.	140	po, cap, SD, w/o KET	28	68	39 (22-54)	77 (56-100)	n.r.	training	[6], Study 2
Food Effect Study, Nguyen et al.	140	po, cap, SD, fasted	47	46	38 (18-55)	76 (49-96)	n.r.	training	[7], Study 1
PPI Effect Study, Nguyen et al.	100	po, tab, SD	22	41	38 (25-50)	72 (56-100)	n.r.	test	[7], Study 2
Liver impairment, Nguyen et al.	60	po, cap SD, healthy	10	100	54 (43-65)	89 (65-107)	n.r.	liver	[8]
Liver impairment, Nguyen et al.	60	po, cap SD, mild impairment	8	100	56 (40-65)	92 (71-112)	n.r.	liver	[8]
Liver impairment, Nguyen et al.	60	po, cap SD moderate impairment	8	100	58 (53-62)	86 (66-104)	n.r.	liver	[8]

BE: bioequivalence, cap: capsule, ig: intragastric, KET: Ketoconazole, n: number of individuals per study, n.r.: not reported, po: per os, RIF: Rifampin, SD: single dose, tab: tablet, w/o: without. Values in brackets given for age, weight, and height are minima and maxima, all po administrations were given to human subjects.

Table S2. Sampling times in minutes for rat and human blood samples in each study.

Study	Sampling Times [hours]	References
PK in rats, Wang et al.	0, 0.0833, 0.5, 0.75, 1, 2, 3, 4, 6, 8, 24, 48	[4]
Phase I Pharmacokinetics Nguyen et al.	0.5, 1, 2,3, 4, 5, 6, 8, 10, 14, 24, 48, 72, 96, 120, 168, 240, 336, 408, 504 h	[5]
Phase I Bioequivalence Nguyen et al.	0.5, 1, 2, 3, 4, 5, 6, 7,8, 10, 14, 24, 48, 72, 96, 120, 168, 240, 288, 336, 408, 504	[5]
DDI Studies Nguyen et al.	0.5, 1, 2, 3, 4, 5, 6, 7, 8, 9, 10, 14, 24, 72, 96, 120, 144, 168, 240, 288, 336, 408, 504	[6]
Food Effect Study Nguyen et al	0.5, 1, 2, 3, 4, 5, 6, 8, 10, 14, 24, 48, 72, 120, 168, 240, 336, 408, 504	[7], Study 1
PPI Effect Study Nguyen et al.	0.5, 1, 2, 3, 4, 5, 6, 8, 10, 14, 24, 48, 72, 120, 168, 240, 336, 408, 504	[7], Study 2
Liver impairment Study, Nguyen et al.	0.5, 1, 2, 3, 4, 5, 8, 14, 24, 48, 72, 96, 120, 168, 240, 288, 336, 432, 504	[9]

DDI: drug-drug interaction; PPI: proton-pump inhibitor.

2. Inspection of Rat and Human Plasma Concentration-time Data

Plasma concentration-time data after intravenous (iv) and intragastric (ig) administration to Sprague-Dawley rats were extracted from literature [4] and plotted dose normalized (**Error! Reference source not found.**) to identify nonlinear properties.

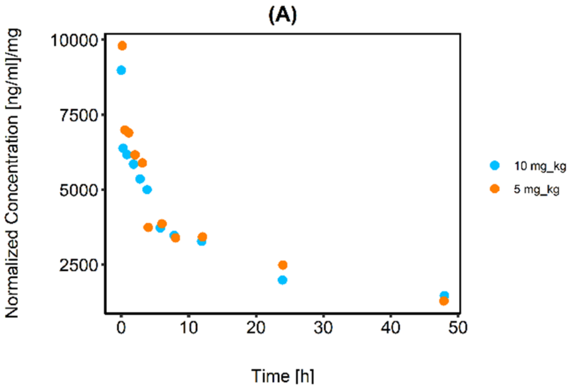

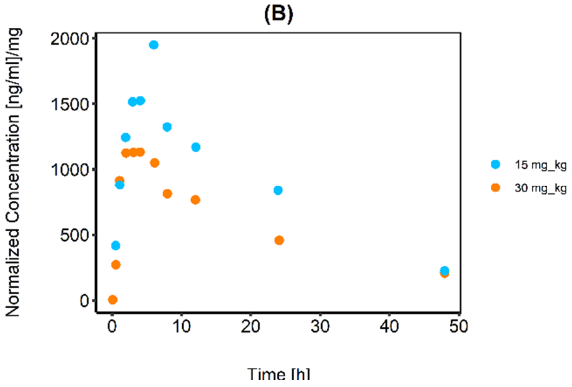

Figure S3. Visual inspection of dose normalized plasma concentration-time curves from 0 to 50 hours for (**A**) iv administration of 5 mg/kg and 10 mg/kg to Sprague-Dawley rats and (**B**) intragastric administration of 15 mg/kg and 30 mg/kg to Sprague-Dawley rats.

For all available data in human healthy subjects, dose normalization was performed on the exposure parameters AUC_{0_inf} (area under the concentration–time curves from the first data point to infinity) and C_{max} (maximum concentrations) (**Error! Reference source not found.**) to verify linear pharmacokinetics and to gain hints for processes like supersaturation or colon absorption. Dose normalized spaghetti plots in linear and semi-logarithmic scale are shown in Figure S5. In contrast to the plotted rat data, no nonlinear PK could be observed in humans after oral administration, which is consistent with the findings of Lacy et al.[3]. They further describe a significantly lower AUC_{0_inf} when CAB is combined with RIF. Higher systemic exposure is also described for mild and moderate liver impairment by the authors. **Error! Reference source not found.** presents these observations graphically. In addition, concentration-time profiles were plotted after administration of CAB tablets (20, 40, 60, 100 and 140 mg), but without dose normalization (**Error! Reference source not found.**). These plots can help to identify existing deposition effects e.g., via binding to proteins or to tissue components. Due to the gradual release of the substance from such a depot, a relatively slow decrease of the plasma concentration at later time points with a long terminal half-life combined with a rapid decrease of the plasma concentration at the beginning would be observed in this case. This effect would be independent of the given dose. As CAB also shows that dichotomy in plasma concentration-time profile, this option was tested, but could not be confirmed as different doses resulted in different plasma concentrations in the second, slowly declining phase. Furthermore, a deposit effect does not automatically explain multiple peaks in plasma concentration-time profiles. As only mean data were available for model development, there might also be a theoretical possibility, that some patients have extraordinary high plasma concentrations after approximately 24 hours, resulting in a general increase of the plasma concentration-time profile at that time. However, because this characteristic peak after 24 h appears in almost all studies, with different study participants, that theory was excluded.

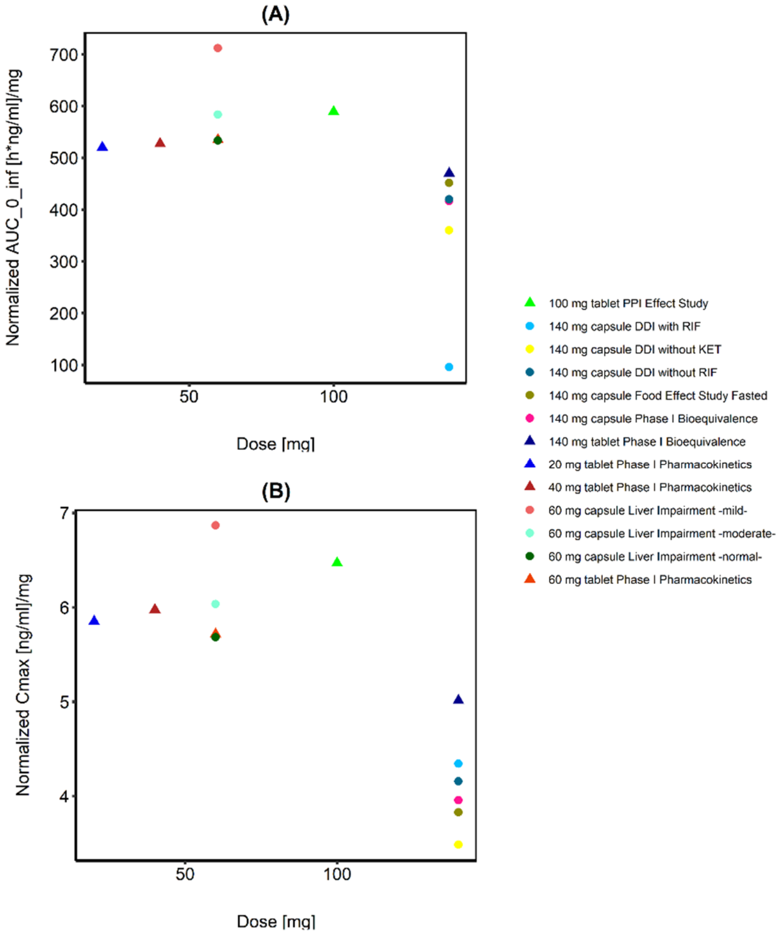

Figure S4. Visual inspection of dose normalized PK parameters versus dose in humans. (**A**) area under the plasma concentration-time curve from time zero to infinity (AUC_{0_inf}), (**B**) maximum observed concentration.

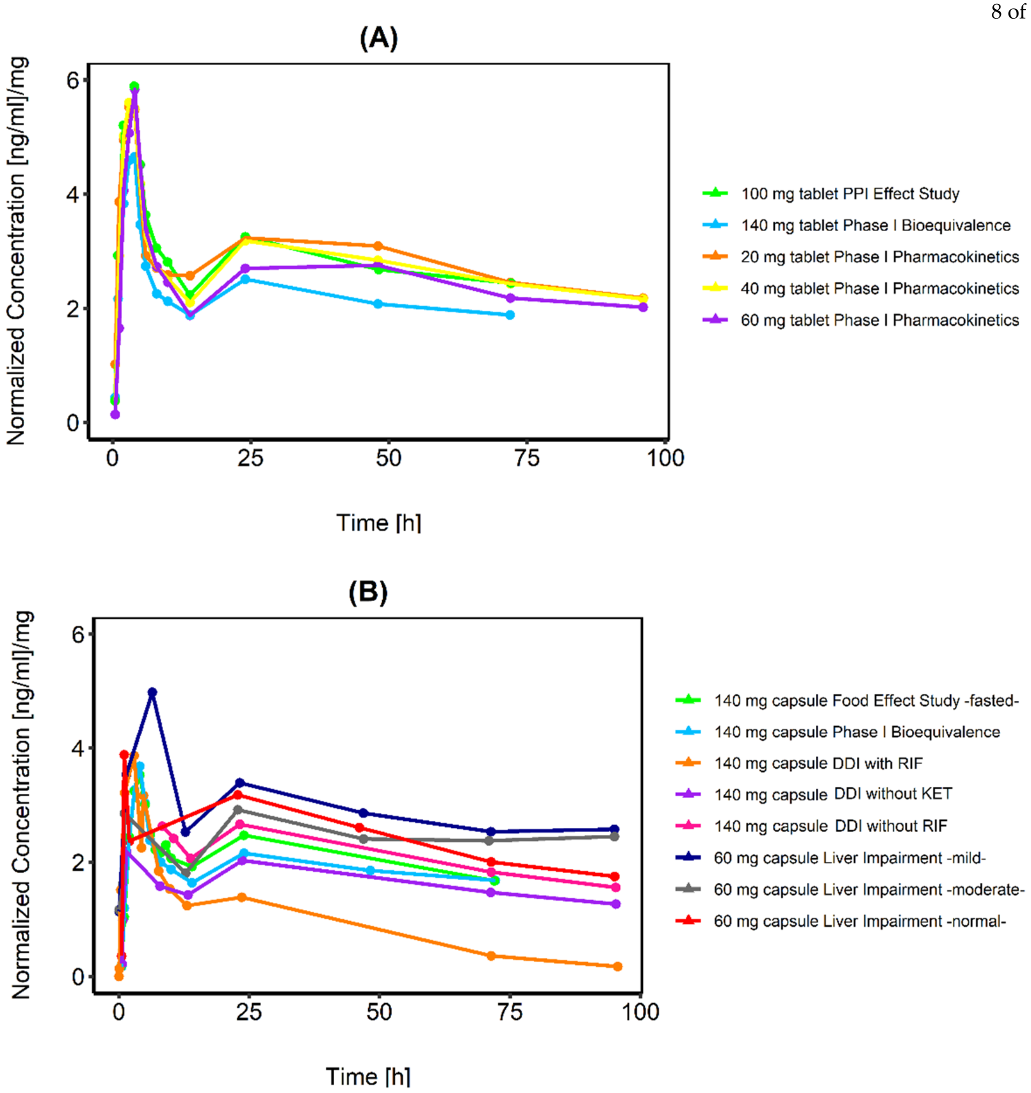

Figure S5. Visual inspection of dose normalized plasma concentration-time curves from 0 to 100 h for (**A**) tablet formulations and (**B**) capsule formulations.

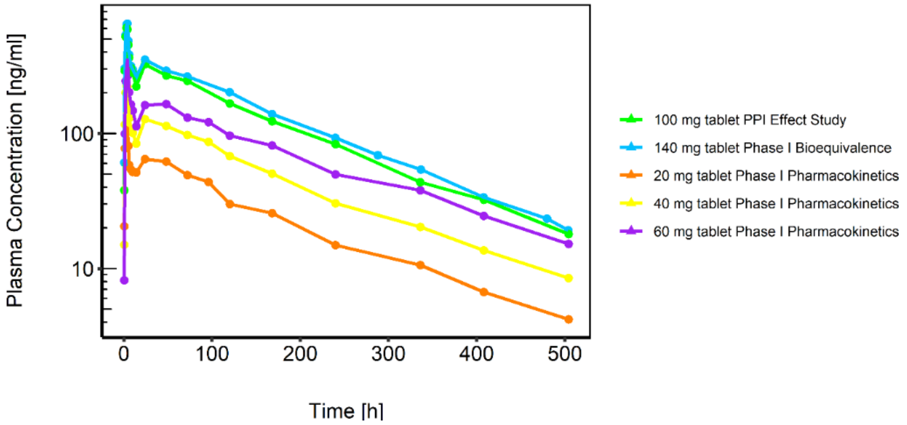

Figure S6. Semi-logarithmic plots of plasma concentration-time profiles after single oral administrations of 20, 40, 60 and 100, respectively 140 mg CAB tablet to healthy humans.

3. Rat Intravenous and Intragastric Simulations

The final parameter used for the rat PBPK model are shown in **Error! Reference source not found.**. The simulated fraction absorbed for ig 30 mg/kg was lower (0.23) compared to ig 15 mg/kg (0.34) and is in agreement with the nonlinear properties and the lower plasma concentration for the higher dose, which was observed in the dose-normalized plasma concentration-time plots and attributed to differences in drug absorption. Besides the graphical check, model evaluation was done through comparison of the predicted vs observed PK parameters C_{max} and AUC_{last} (Area under the concentration time curve from the first to the last data point) as well as through calculation of the mean prediction error (MPE) and mean absolute prediction error (MAPE) to evaluate bias and prediction of the rat model. Both models show a high accuracy illustrated by a low bias (MPE range –6.4% to + 12.2%) and a good precision (MAPE range 18.4–33.8%). A mean relative deviation (MRD) of all predicted plasma concentrations ≤ 2 characterize an adequate model performance and was achieved in all simulations (MRD range 1.23–1.63). **Error! Reference source not found.** summarizes the respective PK parameters C_{max} and AUC_{last} as well as the values for MPE, MAPE and MRD.

Table S3. Summary of the CAB parameters used in the rat PBPK model.

Parameter	Unit	Value Used in PBPK Model	Literature Value [Reference]	Description
MW	[g/mol]	501.50	501.50 [10,11]	Molecular weight
pK_a [base]		6.32	6.32 [12]	Acid dissociation constant
fu_p		0.24	0.24[a] [2]	Fraction unbound in plasma
logP		4.40[b]	5.15 [12]	Lipophilicity
Solubility (pH 6.5)	[10^{-3} mg/mL]	7.72[b]	0.00 [10]	Solubility

$Cl_{hepatic}$	[mL/min/kg]	0.08[b]	--	Total plasma clearance in liver
Partition coefficients		Rodgers and Rowland	[13,14]	Calculation method cell to plasma coefficients
Cellular permeabilities		PKSim® Standard	[15]	Calculation method permeation across cell membranes

[a] based on human plasma protein binding; [b] Model parameters have been estimated through parameter optimization based on the plasma concentrations; -- Value not available.

Table S4. Predicted and observed AUC_{last} and C_{max} values of CAB plasma concentrations in rats. Bias (mean prediction error) and precision (mean absolute prediction error) and mean relative deviation.

**Route / Dose**	**AUC_{last}**			**C_{max}**			**MPE**	**MAPE [%]**	**MRD**
	**Pred [ng*h/mL]**	**Obs [ng*h/mL]**	Pred/Obs	Pred [ng/mL]	Obs [ng/mL]	Pred/Obs			
**iv 5 mg/kg**	**154434.0**	**152843.8**	1.0	14117.0	12392.2	1.1	− 6.4	19.2	1.27
iv 10 mg/kg	309075.4	226769.3	1.4	28232.3	20998.2	1.3	+ 1.9	18.4	1.23
ig 15 mg/kg	141369.9	147716.7	1.0	4681.3	6725.8	0.7	+ 12.2	31.3	1.48
ig 30 mg/kg	237664.6	179824.5	1.3	8019.9	7806.45	1.0	+ 10.4	33.8	1.63

AUC_{last}: Area under the concentration time curve from the first to the last data point, C_{max}: maximum plasma concentration, MAPE: mean absolute prediction error, MPE: mean prediction error, MRD: mean relative deviation, Obs: observed value, Pred: predicted value.

4. Human PBPK Model Evaluation

In addition to the plots shown in the main manuscript, semi-logarithmic plots of population predictions compared to observed plasma concentration-time profiles are shown in **Error! Reference source not found.**. **Error! Reference source not found.** shows the predicted vs. observed AUC_{last} C_{max} values of all studies. Mean predicted and observed AUC_{last} and C_{max} values, model bias (mean prediction error), model precision (mean absolute prediction error) and mean relative deviation (MRD) are listed in **Error! Reference source not found.** and **Error! Reference source not found.**. Results of the local sensitivity analysis, which was made based on the simulation of the 140 mg CAB capsule administration, are demonstrated in **Error! Reference source not found.**.

Semi-logarithmic plots

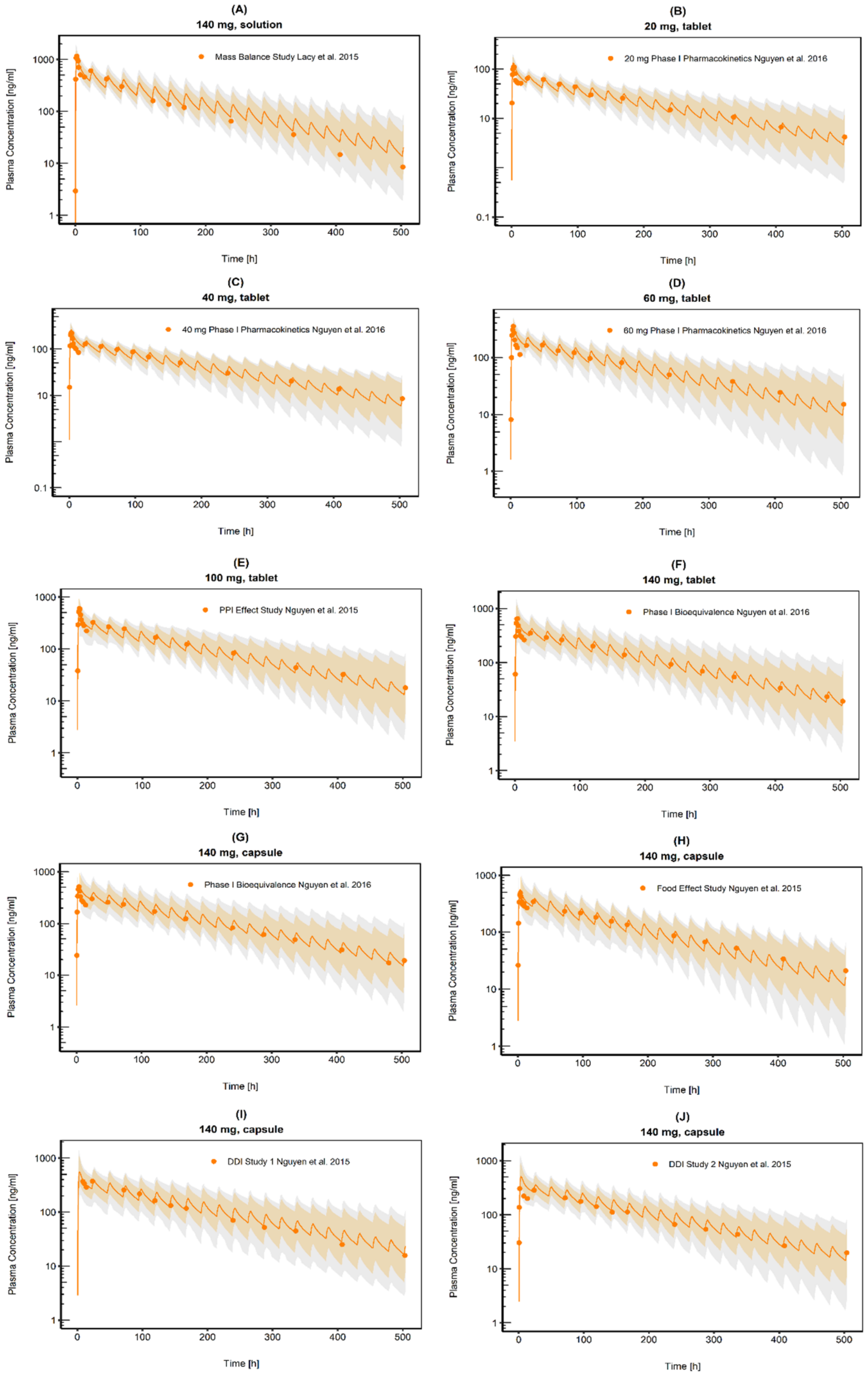
(A)
140 mg, solution
Mass Balance Study Lacy et al. 2015
(B)
20 mg, tablet
20 mg Phase I Pharmacokinetics Nguyen et al. 2016
(C)
40 mg, tablet
40 mg Phase I Pharmacokinetics Nguyen et al. 2016
(D)
60 mg, tablet
60 mg Phase I Pharmacokinetics Nguyen et al. 2016
(E)
100 mg, tablet
PPI Effect Study Nguyen et al. 2015
(F)
140 mg, tablet
Phase I Bioequivalence Nguyen et al. 2016
(G)
140 mg, capsule
Phase I Bioequivalence Nguyen et al. 2016
(H)
140 mg, capsule
Food Effect Study Nguyen et al. 2015
(I)
140 mg, capsule
DDI Study 1 Nguyen et al. 2015
(J)
140 mg, capsule
DDI Study 2 Nguyen et al. 2015
Plasma Concentration [ng/ml]
Time [h]

Figure S7. CAB plasma concentration-time profiles (semi-logarithmic). Observed data are shown as orange circles. Population simulation (n = 100) geometric means are shown as orange lines; the shaded orange areas represent the predicted population geometric SD. The shaded grey areas represent the 5 to 95% prediction interval.

Goodness-of-fit plots for AUC and Cmax

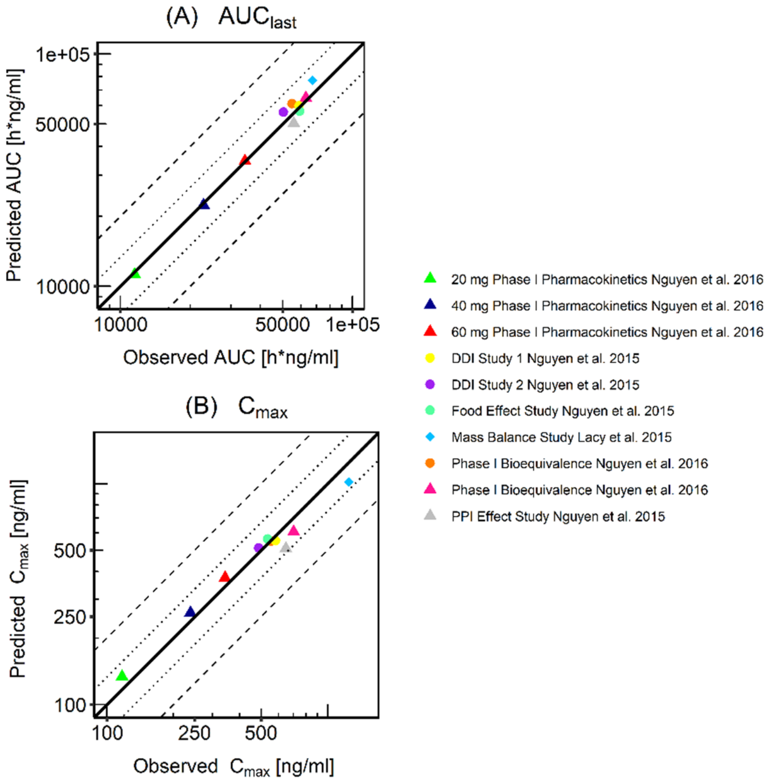

Figure S8. Goodness-of-fit plots for the predicted versus observed (**A)** AUC_{last} and (**B**) C_{max}. Tablet formulations are represented by triangles, capsule formulations are represented by dots, the solution is represented by diamonds. In each plot, the black solid line represents the line of identity; dashed black lines represent a twofold deviation; dotted black lines represent a 1.25-fold deviation.

Sensitivity Analysis

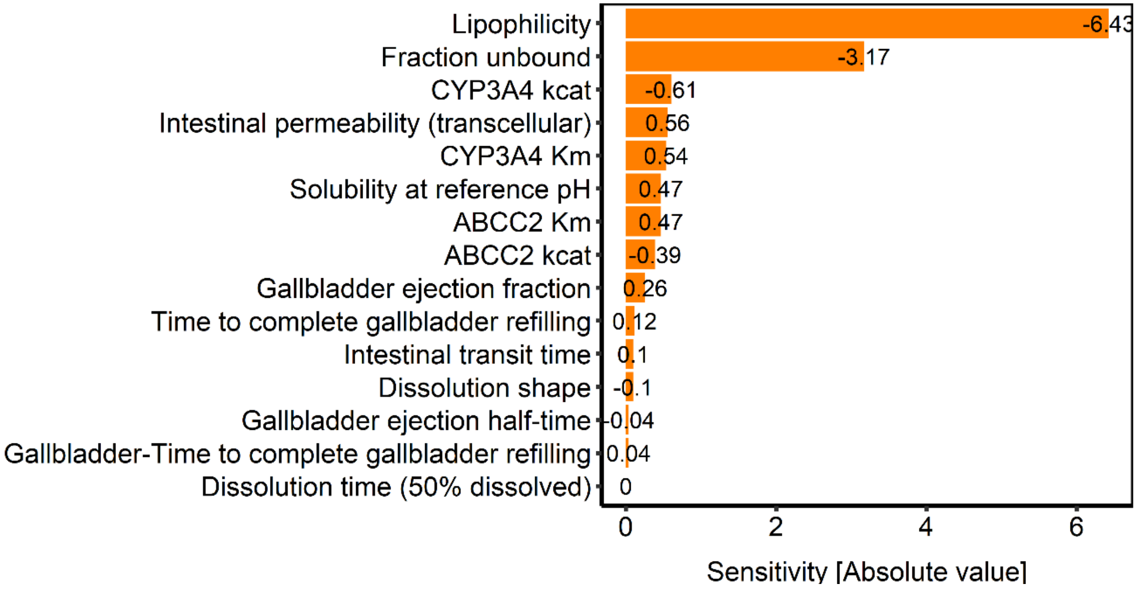

Figure S9. Sensitivity analysis for parameters which were estimated during the model development or which might have an impact due to calculation methods in PK-Sim®. Sensitivity was measured as the relative change of AUC_{last} of a 140 mg CAB capsule single dose administration. Variation range was 10.0 with maximum number of steps= 9. *ABCC2:* MRP2 coding gene, *kcat*: katalytic rate constant, *Km:* Michaelis-Menten constant.

Table S5. Mean predicted and observed pharmacokinetic parameters of CAB after oral single dose of 20, 40, 60, 100 and 140 mg CAB tablet, capsule or solution in healthy volunteers.

Dose, Formulation	AUC_{last}			C_{max}			Reference
	Pred [ng*h/mL]	Obs [ng*h/mL]	Pred/Obs	Pred [ng/mL]	Obs [ng/mL]	Pred/Obs	
20 mg, tablet	11245.2	11508.4	1.0	133.7	117.0	1.1	[5]
40 mg, tablet	22636.5	22781.5	1.0	260.2	239.0	1.1	[5]
60 mg, tablet	34579.8	34376.0	1.0	274.9	343.0	1.1	[5]
140 mg, tablet	64750.2	62895.7	1.0	604.8	702.0	0.9	[5]
140 mg, cap	61111.8	54897.1	1.1	541.5	554.0	1.0	[5]
140 mg, cap	59738.1	58800.0[a]	1.0	551.2	582.0	1.0	[6] Study 1
140 mg, cap	56190.1	50400.0[a]	1.1	510.9	488.0	1.1	[6] Study 2
140 mg, cap	56725.8	59200.0	1.0	561.4	536.0	1.1	[7] Study 1
100 mg, tablet	50274.5	55800.0	0.9	507.5	647.0	0.8	[7] Study 2

140 mg, solution	77017.6	67200.0	1.2	1018.7	1250.0	0.8	[2]

AUC_{last}: Area under the concentration time curve from the first to the last data point, cap: capsule, C_{max}: maximum plasma concentration, Obs: observed value, Pred: predicted value

[a] reported values are area under the concentration time curve from the first data point to infinity.

Table S6. Bias (mean prediction error), precision (mean absolute prediction error) and mean relative deviation (MRD).

Dose, Formulation	MPE	MAPE	MRD	Reference
20 mg, tablet	+ 14.1	24.3	1.32	[5]
40 mg, tablet	+ 20.8	21.7	1.28	[5]
60 mg, tablet	+ 37.2	39.9	1.51	[5]
140 mg, tablet	+ 9.7	18.7	1.24	[5]
140 mg, cap	+ 17.2	30.1	1.75	[5]
140 mg, cap	+ 16.4	19.4	1.22	[6] Study 1
140 mg, cap	+ 10.8	34.9	1.68	[6] Study 2
140 mg, cap	+ 6.8	21.4	1.66	[7] Study 1
100 mg, tablet	- 4.7	13.9	1.88	[7] Study 2
140 mg, solution	+ 43.0	53.3	1.67	[2]

cap: capsule, MPE: mean prediction error, MAPE: mean absolute prediction error, *MRD:* mean relative deviation.

5. Simulations of DDI between CAB and RIF

Table S7. Comparison of average CAB steady state plasma concentrations (C_{ss}) after different CAB and RIF administration schemes to evaluate CAB RIF DDI influence on plasma exposure.

Administration Scheme	CAB Formulation	CAB C_{ss} (ng/mL)	Ratio CAB alone/ CAB+RIF
60 mg CAB	tablet	1197.44	0.67
80 mg CAB + 600 mg RIF	tablet	394.74	
140 mg CAB	capsule	1576.68	0.66
180 mg CAB + 600 mg RIF	capsule	532.41	

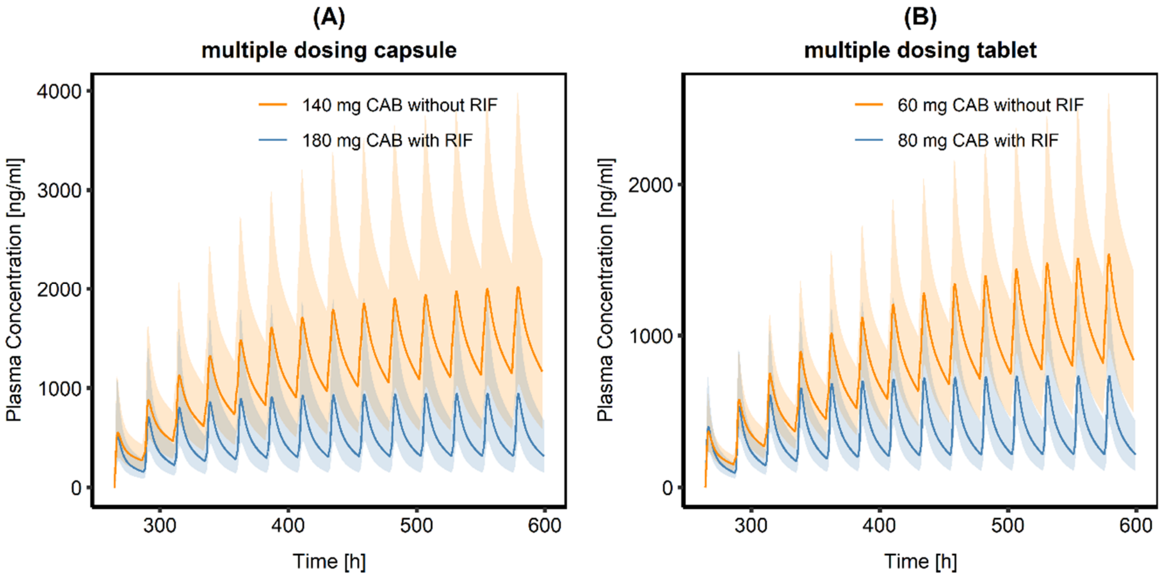

Figure S10. Population simulations (*n* = 100) of CAB steady state plasma concentration-time profiles. (**A**) the orange respectively blue line represents the population simulations geometric mean of the predicted plasma concentration after administration of 140 mg CAB as capsules alone (orange line) or 180 mg CAB as capsules with co-administration of 600 mg RIF (blue line). (**B**) the orange respectively blue line represents the population simulations geometric mean of the predicted plasma concentration after administration of 60 mg CAB in the form of tablets alone (orange line) or 80 mg CAB tablets with co-administration of 600 mg RIF (blue line). CAB and RIF were administered once daily in each case. CAB administration started on day 11 to account for the run-in period of RIF administration alone until reaching a RIF steady state concentration. The shaded areas represent the predicted population geometric SD in each case.

6. Investigation of Hepatic Impairment on CAB Plasma Exposure

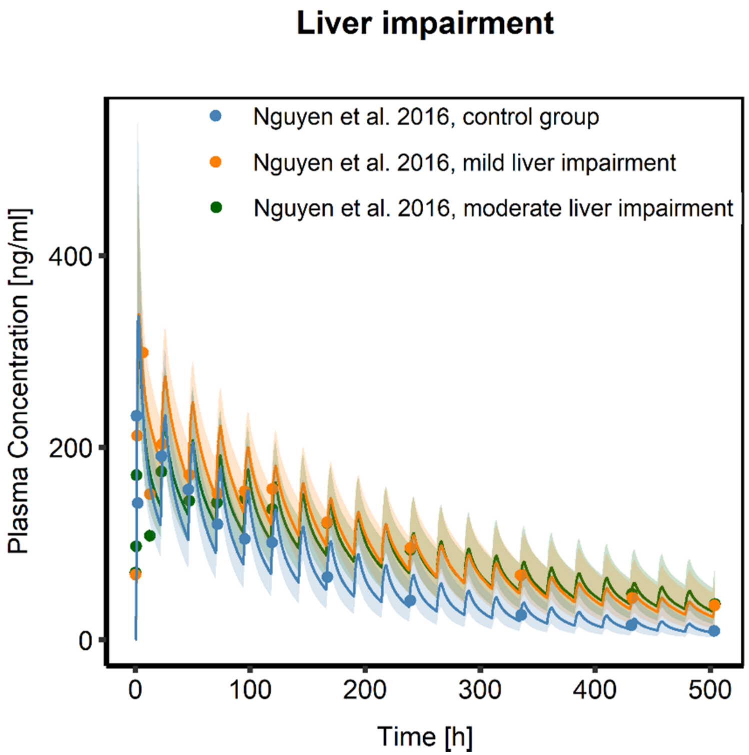

Figure S11. Comparison of simulated plasma concentration-time profiles after a single administration of 60 mg CAB capsule to a healthy control group and a mild and moderate liver impaired population; *n* = 100 in each case. Blue, orange and green dots represent the observed plasma concentation in the respective group; the blue, orange and green line indicate the population simulations geometric mean of the predicted plasma concentration; the shaded areas represent the predicted population geometric SD.

1. Neul, C.; Schaeffeler, E.; Sparreboom, A.; Laufer, S.; Schwab, M.; Nies, A.T. Impact of Membrane Drug Transporters on Resistance to Small-Molecule Tyrosine Kinase Inhibitors. *Trends Pharmacol Sci* **2016**, *37*, 904-932.

2. Lacy, S.; Hsu, B.; Miles, D.; Aftab, D.; Wang, R.; Nguyen, L. Metabolism and Disposition of Cabozantinib in Healthy Male Volunteers and Pharmacologic Characterization of Its Major Metabolites. *Drug Metab Dispos* **2015**, *43*, 1190-1207.

3. Lacy, S.A.; Miles, D.R.; Nguyen, L.T. Clinical Pharmacokinetics and Pharmacodynamics of Cabozantinib. *Clin Pharmacokinet* **2017**, *56*, 477-491.

4. Wang, X.; Wang, S.; Lin, F.; Zhang, Q.; Chen, H.; Wang, X.; Wen, C.; Ma, J.; Hu, L. Pharmacokinetics and tissue distribution model of cabozantinib in rat determined by UPLC-MS/MS. *J Chromatogr B Analyt Technol Biomed Life Sci* **2015**, *983-984*, 125-131, doi:10.1016/j.jchromb.2015.01.020.

5. Nguyen, L.; Benrimoh, N.; Xie, Y.; Offman, E.; Lacy, S. Pharmacokinetics of cabozantinib tablet and capsule formulations in healthy adults. *Anti-Cancer Drugs* **2016**, *27*, 669-678.

6. Nguyen, L.; Holland, J.; Miles, D.; Engel, C.; Benrimoh, N.; O'Reilly, T.; Lacy, S. Pharmacokinetic (PK) drug interaction studies of cabozantinib: Effect of CYP3A inducer rifampin and inhibitor ketoconazole on cabozantinib plasma PK and effect of cabozantinib on CYP2C8 probe substrate rosiglitazone plasma PK. *J Clin Pharmacol* **2015**, *55*, 1012-1023.

7. Nguyen, L.; Holland, J.; Mamelok, R.; Laberge, M.K.; Grenier, J.; Swearingen, D.; Armas, D.; Lacy, S. Evaluation of the effect of food and gastric pH on the single-dose pharmacokinetics of cabozantinib in healthy adult subjects. *J Clin Pharmacol* **2015**, *55*, 1293-1302.

8. Nguyen, L.; Holland, J.; Ramies, D.; Mamelok, R.; Benrimoh, N.; Ciric, S.; Marbury, T.; Preston, R.A.; Heuman, D.M.; Gavis, E. Effect of renal and hepatic impairment on the pharmacokinetics of cabozantinib. *The Journal of Clinical Pharmacology* **2016**, *56*, 1130-1140.

9. Nguyen, L.; Holland, J.; Ramies, D.; Mamelok, R.; Benrimoh, N.; Ciric, S.; Marbury, T.; Preston, R.A.; Heuman, D.M.; Gavis, E.; et al. Effect of Renal and Hepatic Impairment on the Pharmacokinetics of Cabozantinib. *J Clin Pharmacol* **2016**, *56*, 1130-1140, doi:10.1002/jcph.714.

10. U.S. Food and Drug Administration. Cometriq (cabozantinib) Capsules. Chemistry Review(s) 2012; https://www.accessdata.fda.gov/drugsatfda_docs/nda/2012/203756Orig1s000ChemR.pdf (accessed on 2020 Jan 15).

11. U.S. Food and Drug Administration. CABOMETYX (cabozantinib) Tablets. Chemistry Review(s) 2015; https://www.accessdata.fda.gov/drugsatfda_docs/nda/2016/208692Orig1s000ChemR.pdf (accessed on 2020 Jan 15).

12. Ipsen Biopharmaceuticals Canada Inc. CABOMETYX Product Monograph. 2019; https://ipsen.com/websites/IPSENCOM-PROD/wp-content/uploads/sites/18/2019/11/21094828/Cabometyx-PM-EN-07Nov2019.pdf (accessed on 2020 Jan 15).

13. Rodgers, T.; Leahy, D.; Rowland, M. Physiologically based pharmacokinetic modeling 1: predicting the tissue distribution of moderate-to-strong bases. *J Pharm Sci* **2005**, *94*, 1259-1276.

14. Rodgers, T.; Rowland, M. Physiologically based pharmacokinetic modelling 2: predicting the tissue distribution of acids, very weak bases, neutrals and zwitterions. *J Pharm Sci* **2006**, *95*, 1238-1257.

15. Open Systems Pharmacology. PK-Sim® software manual. Available online: https://docs.open-systems-pharmacology.org/ (accessed on 2019 Sep 26).
